# Exercise ameliorates osteopenia in mice via intestinal microbial-mediated bile acid metabolism pathway

**DOI:** 10.7150/thno.104186

**Published:** 2025-01-02

**Authors:** Congcong Yu, Rongtai Sun, Wentao Yang, Tianyuan Gu, Xiaozhang Ying, Lin Ye, Yang Zheng, Shunwu Fan, Xiangjun Zeng, Shasha Yao

**Affiliations:** 1 Department of Orthopaedic Surgery, Sir Run Run Shaw Hospital School of Medicine, Zhejiang University, Hangzhou, Zhejiang 310016, China.; 2 Key Laboratory of Musculoskeletal System Degeneration and Regeneration, Translational Research of Zhejiang Province Hangzhou, Zhejiang 310016, China.; 3 Zhejiang Hospital of Integrated Traditional Chinese and Western Medicine, Hangzhou, Zhejiang 310016, China.; 4 Research Institute of Orthopedics, The Affiliated Jiangnan Hospital of Zhejiang Chinese Medical University, Hangzhou, Zhejiang 310053, China.; 5 Bone Marrow Transplantation Center of the First Affiliated Hospital & Liangzhu Laboratory, Zhejiang University School of Medicine, Hangzhou, Zhejiang 311100, China.

**Keywords:** osteoporosis, gut microbiome, exercise, bile acid, apelin

## Abstract

**Rationale:** Physical exercise is essential for skeletal integrity and bone health. The gut microbiome, as a pivotal modulator of overall physiologic states, is closely associated with skeletal homeostasis and bone metabolism. However, the potential role of intestinal microbiota in the exercise-mediated bone gain remains unclear.

**Methods:** We conducted microbiota depletion and fecal microbiota transplantation (FMT) in ovariectomy (OVX) mice and aged mice to investigate whether the transfer of gut ecological traits could confer the exercise-induced bone protective effects. The study analyzed the gut microbiota and metabolic profiles via 16S rRNA gene sequencing and LC-MS untargeted metabolomics to identify key microbial communities and metabolites responsible for bone protection. Transcriptome sequencing and RNA interference were employed to explore the molecular mechanisms.

**Results:** We found that gut microbiota depletion hindered the osteogenic benefits of exercise, and FMT from exercised osteoporotic mice effectively mitigated osteopenia. Comprehensive profiling of the microbiome and metabolome revealed that the exercise-matched FMT reshaped intestinal microecology and metabolic landscape. Notably, alterations in bile acid metabolism, specifically the enrichment of taurine and ursodeoxycholic acid, mediated the protective effects on bone mass. Mechanistically, FMT from exercised mice activated the apelin signaling pathway and restored the bone-fat balance in recipient MSCs.

**Conclusion:** Our study underscored the important role of the microbiota-metabolic axis in the exercise-mediated bone gain, heralding a potential breakthrough in the treatment of osteoporosis.

## Introduction

Osteoporosis, characterized by low bone mass and trabecular deterioration, is a prevalent bone disorder in the elderly, especially in postmenopausal women 1. The imbalance of bone metabolism and increased levels of inflammatory factors caused by estrogen deficiency or aging are closely associated with the decline of bone mass 2, 3. Although certain medications, such as bone resorption inhibitors and calcium supplements, have been proven to effectively alleviate osteoporosis, there is still a need for more ideal interventions to improve bone health and prevent osteoporosis 4, 5. It is well recognized that engaging in physical activity is essential for maintaining bone mass and preventing bone diseases 6, 7. In a randomized controlled trial, postmenopausal women with decreased bone mass have shown significant improvements in bone strength through high-intensity resistance and impact exercises 8. Consistently, in ovariectomy (OVX)-induced osteoporosis mouse models, exercise serves as a potent defender against bone loss while facilitating angiogenesis through the augmentation of extracellular vesicle miRNAs levels in the bloodstream 9. However, the detailed mechanisms of the exercise-mediated protective effects on skeletal system remain largely elusive. A deeper understanding of these mechanisms is crucial for developing innovative approaches to the prevention and treatment of osteoporosis.

The gut microbiota, an intricate community of microorganisms, has emerged as an important regulator of the host's physiological processes across both health and disease states 10-12. Of note, accumulating evidence has implicated a tight connection between intestinal microbiota and bone homeostasis 13, 14. In both sex steroid-deficient and sex steroid-normal models, germ-free mice exhibit a suppression of osteoclastic activity, an enhancement in bone mass, and an improvement of trabecular bone when compared to conventionally raised mice 15, 16. Additionally, fecal microbiota transplantation (FMT) from young donors, rather than from old donors, can potently stimulate osteoblastic activity and inhibit osteoclast formation, thereby lessening the osteoporotic phenotype by facilitating the accumulation of extracellular vesicles within bone tissue 17. Moreover, some probiotics and gut microbiota-derived metabolites have been shown to correct the imbalanced bone metabolism and prevent bone loss 17, 18.

Nevertheless, it remains unclear whether the impact of exercise on bone is mediated by the gut microbiota, and which specific regulatory factors within the gut are crucial underlying the therapeutic benefits of exercise in the treatment of osteopenia. Here, we emphasized the importance of gut microbiota in the exercise-induced beneficial effects on bone mass by employing antibiotic-induced models and FMT models. Furthermore, 16S rRNA gene microbiota sequencing, RNA sequencing (RNA-Seq), and liquid chromatography-mass spectrometry (LC-MS) untargeted metabolomic analysis were carried out to delve into the mechanisms responsible for bone responses to exercise. Considering the profound role of gut microbiota in regulating osteogenic capacity, our study holds promise for advancing the understanding and treatment of metabolic bone disease.

## Methods

### Animals

Female mice at 4 weeks old and male mice at 17 months old were purchased from Hangzhou Medical College (Zhejiang, China), then maintained under a 12-hour light/dark cycle at 25 °C with ad libitum access to sterile food and water. After one month of acclimatization, the animals were used for experimentation. All animal experiments were approved by the Institutional Animal Care and Use Committee of the Zhejiang Center of Laboratory Animals. Female mice at 8 weeks old underwent bilateral OVX surgery, which involved ligating and removing both ovaries through a dorsal incision 13. The success of the operation was validated by vaginal cytology. Mice in the Sham group received corresponding sham surgery. Following a week of postoperative convalescence, the mice commenced an 8-week treadmill training regimen. Besides, 18-month-old aged male mice were also enrolled in the treadmill exercise. The body weight and food intake of the mice were recorded daily. Upon completion of the experiments, the mice were weighed, anesthetized, and various biological samples were meticulously procured for analysis prior to their euthanization.

### Treadmill exercise

The treadmill training was performed as previously described 19. An eight-lane animal treadmill (NJKEWBIO, Nanjing, China) was used for the exercise training. Mice initiated training with daily 60-minute sessions at 6 m/min, incrementing the speed by 1 m/min daily until reaching a consistent 12 m/min for 60 minutes. This routine was continued daily throughout the 8-week exercise program.

### Bone microcomputed tomography (µCT) imaging

The mouse tibias were isolated and completely cleared off muscle tissue. The tibias were examined with a µCT system (Skyscan 1275, Bruker, Belgium) at 9 µm voxel size. The growth plate was selected as a reference, and the grayscale range was set to 70 offset, and 100 consecutive images of μCT scans were used for bone trabecular region selection. The grayscale range was set to 150 offset, and 100 consecutive images were used for cortical bone region selection. Image reconstruction was performed using the data viewer and CTAn softwares for region of interest selection and quantitative data. Besides, 3D images were obtained using the CTvox software. Measurements were obtained, including bone mineral density (BMD), bone volume fraction (BV/TV), trabecular number (Tb. N), trabecular thickness (Tb. Th), trabecular separation (Tb. Sp), cortical thickness (Ct. Th), and cortical bone area fraction (Ct. Ar/Tt. Ar).

### Biomechanical analysis

Mouse femurs were dissected, defatted, and then preserved in 75% ethanol solution. For the three-points bending test to address the biomechanical parameters, femurs were placed with a span of 10 mm and load was applied to the midpoint of the shaft (creating a three-points bending). Mechanical resistance to failure was measured using an electronic tensile tester (CMT-6104, SANS, China) with actuator displaced at 2 mm/min. Ultimate force and yield point were further calculated.

### Bone histomorphometry

For histomorphometric analysis, the tibias were fixed in 4% paraformaldehyde for 48 hours and then decalcified in 10% EDTA (Aladdin, Shanghai, China) with continuous shaking for one week. Subsequently, the softened tibias were embedded in paraffin and sectioned. The morphology of the proximal trabecular bone was examined using Hematoxylin and eosin (H&E) staining (Solarbio, Beijing, China). Immunohistochemistry (IHC) staining was performed with osteocalcin (OCN) antibody (Bioss, USA) and apelin antibody (Proteintech, China) by a SP Rabbit & Mouse HRP kit (CWBIO, China) to quantify osteoblasts on the trabecular surface and assess apelin expression within the bone tissue, respectively. The number of osteoclasts on the trabecular surface was assessed by tartrate-resistant acid phosphatase (TRAP) staining (Solarbio, Beijing, China).

To assess dynamic bone formation, mice were intraperitoneally injected with a 0.1% calcein (Sigma-Aldrich, Germany) PBS solution 9 and 3 days prior to euthanasia. The tibias were harvested, dehydrated through gradient concentrations of ethanol, and embedded in methyl methacrylate (Sigma-Aldrich, Germany). Undecalcified bone sections with a thickness of 10 μm were prepared and observed under a fluorescence microscope (DMi8, Leica, Germany) for the calcein double labeling. The mineral apposition rates (MAR) of the trabecular and cortical bone were quantified and calculated.

### Serum biochemical analysis

Blood was drawn via ocular puncture from anesthetized mice, and serum was isolated by centrifugation at 4 °C at 4000 rpm. Commercial ELISA kits were employed to quantify serum levels of OCN (Abcam, USA), C-terminal telopeptides of type I collagen (CTX-1) (Reddot Biotech, Canada), and apelin (Sigma-Aldrich, Germany). Additionally, the levels of lipopolysaccharide (LPS) and inflammatory cytokines, including interleukin 1β (IL1β), interleukin 6 (IL6), interleukin 17 (IL17), and tumor necrosis factor α (TNFα), were assessed by ABclonal Technology (Wuhan, China).

### Antibiotic treatment

The antibiotic cocktail solution was prepared by mixing ampicillin (0.25 mg/mL), metronidazole (0.25 mg/mL), neomycin (0.25 mg/mL), and vancomycin (0.125 mg/mL) in autoclaved water 20. These antibiotics were purchased from Sigma-Aldrich, Germany. The mice with gut microbiota depletion were administered this cocktail for one week prior to initiating treadmill training, followed by a switch to standard autoclaved water. All other experimental animals received only standard autoclaved water throughout the experimental period.

### 16S rRNA sequencing and LC-MS untargeted metabolomics

Mice were transferred to separate, clean, and empty cages for feces collection. Fresh feces samples were preserved in cryotubes, frozen in liquid nitrogen, and stored at -80 °C. The 16S rRNA sequencing and LC-MS untargeted metabolomics of feces samples were carried out by LC-Bio Technology Co., Ltd (Hangzhou, China). The sequencing of gut microbiome was performed according to the manufacturer's recommendations, and the relative abundance in bacterial taxonomy, alpha diversity, and beta diversity were determined as previously depicted 12. The detection and analysis of metabolomics were conducted as previously described 21.

### FMT

Before microbiota transplantation, the original gut microbiota was depleted through antibiotic treatment. Sterile fecal samples were procured daily from mice engaged in 8 weeks of continuous exercise or from sedentary controls. Fresh fecal pellets from the donors, weighing 100 mg, were added to 1 mL of sterile PBS and homogenized. After a brief centrifugation (300 g, 30 s), the supernatant was immediately gavaged at 100 μL to the respective recipients. This process was continued for 8 weeks.

### Cell culture

The femurs and tibias of mice were collected, and the epiphyses were removed to flush out the marrow with αMEM medium (Gibco, USA) containing 15% FBS (Gibco, USA). The marrow flush was repeatedly pipetted to obtain a single-cell suspension, which was then centrifuged at 1000 rpm for 5 min. The cell pellet was resuspended in αMEM medium with 15% FBS and cultured in a 37 °C, 5% CO_2_ incubator. Medium was refreshed every 2-3 days, with repeated PBS washes. After 7 days, MSCs were isolated and prepared for subsequent experiments.

### Reverse transcription-quantitative polymerase chain reaction (RT-qPCR)

For cell samples and small intestine tissues, total RNA was extracted using a RNA extraction kit (AGbio, China), and the concentration and purity of RNA were determined by UV absorption method (Thermo Fisher Scientific, NanoDrop® ND-2000). Reverse transcription was performed on extracted RNA using a reverse transcription kit (AGbio, China), and qPCR was carried out through a SYBR® Green Premix Pro Taq HS qPCR Kit (AGbio, China) on a real-time fluorescence quantitative PCR system (QuantStudio 6 Flex, Applied Biosystems, USA). The primer sequences used were shown in Table S1.

### Osteogenic potential assessments

MSCs extracted from various groups of mice were further cultured in an osteogenic medium (αMEM containing 10% FBS, 50 mg/mL ascorbic acid, and 10 mM β-glycerophosphate) to assess osteogenic potential. Following a 7-day incubation period, alkaline phosphatase (ALP) activity of cells was quantified using an ALP staining kit (Yeasen, Shanghai, China) and an ALP activity detection kit (Yeasen, Shanghai, China). Subsequently, after a 14-day culture, the formation of mineralization nodules was analyzed using an ARS staining solution (Solarbio, Beijing, China). The red-stained mineralized deposits were dissolved in a 0.5 M HCl solution (Sinopharm, China) containing 5% SDS (Aladdin, Shanghai, China), and the absorbance was measured at 405 nm.

### RNA-Seq

MSCs isolated from different groups were used for RNA-Seq. The TruSeq™ RNA sample preparation kit (Illumina, USA) was used to prepare the RNA-seq libraries. The library quality was verified using an Agilent 2100 Bioanalyzer (Agilent, USA). Sequencing was performed on the Illumina HiSeq 4000 sequencing platforms at Majorbio Biotech Co., Ltd (Shanghai, China). A corrected *P*-value (Benjamini & Hochberg method) of 0.05 and an absolute foldchange of 2 were set as the threshold for differentially expressed genes (DEGs). Gene ontology (GO) function and Kyoto Encyclopedia of Genes and Genomes (KEGG) pathway enrichment analysis were performed by clusterProfiler package v3.18.0 of R software. FDR < 0.05 was considered significant.

### Apelin signaling pathway inhibition

The apelin signaling pathway was suppressed using the apelin receptor antagonist ML221 (TargetMol, USA), wherein mice were administered ML221 (150 μg/kg) via tail vein injections three times a week for 8 weeks 22. The same vehicle (primarily PBS) was injected as a control.

The knockdown of the *Apln* gene *in vivo* was performed using an adeno-associated virus (AAV) vector expressing the shRNA to *Apln* and EGFP. AAV-Ctrl shRNA or AAV-Apln shRNA (1 × 10^11^ genome copies/mL) was injected into the mice via the tail vein (100 µL per mouse). Two weeks later, the EGFP fluorescence in the mice was detected by an IVIS Imaging System 200 (Caliper Life Sciences, USA), and the mice were used for subsequent experiments.

### Quantification of inflammatory cytokines in bone marrow (BM)

The femurs and tibias from mice were harvested and the marrow cavities were flushed with 1 mL of PBS per mouse. The resulting wash was centrifuged at 4 °C at 13,000 rpm for 10 min to yield the supernatant, which was then stored at -80 °C. The assessments of inflammatory cytokines (IL1β, IL6, IL17, TNFα, and IFNγ) in the BM supernatant were performed by ABclonal Technology (Wuhan, China).

### Intestinal histopathology

The intestinal tissues (small intestine and colon) from mice were collected and promptly cleansed with PBS to eliminate luminal contents. The tissues were then immersed in 4% paraformaldehyde for 48 hours for fixation, followed by paraffin embedding and sectioning. The sections of small intestine were stained using an AB-PAS staining kit (Solarbio, Beijing, China) to visualize the small intestinal structure and highlight goblet cells. Representative regions were selected for quantification of goblet cells. The sections of colon were stained using a H&E staining kit. Immunofluorescent staining of intestinal tissues was performed using Muc2 antibody (ABclonal Technology, China) and Tjp1 antibody (ABclonal Technology, China), and the cell nuclei were counterstained with DAPI. The fluorescence intensity was observed and recorded under a fluorescence microscope.

### Intestinal permeability test

To evaluate intestinal permeability, mice were orally gavaged with 300 μL fluorescein isothiocyanate (FITC)-dextran (Sigma-Aldrich, Germany) solution in PBS (100 mg/mL). After 4 hours, serum samples were obtained and the fluorescence intensity was measured at excitation and emission wavelengths of 470 nm and 520 nm, respectively.

### Administration of specific bacterial taxa

Prior to the administration of specific bacteria, OVX mice were initially subjected to a one-week regimen of oral antibiotic cocktail solution to deplete the gut microbiota. Subsequently, the mice were gavaged every two days with 200 μL of reduced PBS containing microbiota (5 × 10^8^ CFU/mL), and the control group mice were administered an equivalent volume of reduced PBS. After an 8-week duration, the mice were euthanized for further analysis and evaluation. *Lachnospira* (DSM #107526), *Odoribacter* (DSM #20712), *Anaerotruncus* (DSM #28734), and *Pseudoflavonifractor* (DSM #23940) were purchased from DSMZ (Germany).

### Administration of metabolites in mice

Mice were orally gavaged with different metabolites, including taurine (Tau), ursodeoxycholic acid (UDCA), linoleic acid (LA), all-trans-retinoic acid (ATRA), and 3-hydroxyanthranilic acid (3HAA) (Sigma-Aldrich, Germany). Mice were dosed orally with 200 mg/kg of metabolites daily over an 8-week period. Mice in the control group received equivalent volumes of PBS by the same gavage method.

### Statistical analysis

GraphPad Prism software was used for statistical analysis. Data in graphs are shown as means ± SEM. For comparisons between two groups, two-tailed unpaired *t* test was used to determine the statistical significance. For comparisons among three or more groups, one-way ANOVA followed by Bonferroni's multiple comparisons test was used to determine the statistical significance. Differences between groups with p < 0.05 were considered significant (*p < 0.05, **p < 0.05, ***p < 0.01).

## Results

### Antibiotic treatment inhibited the protective effects of exercise against OVX-induced bone loss

**Figure 1:**
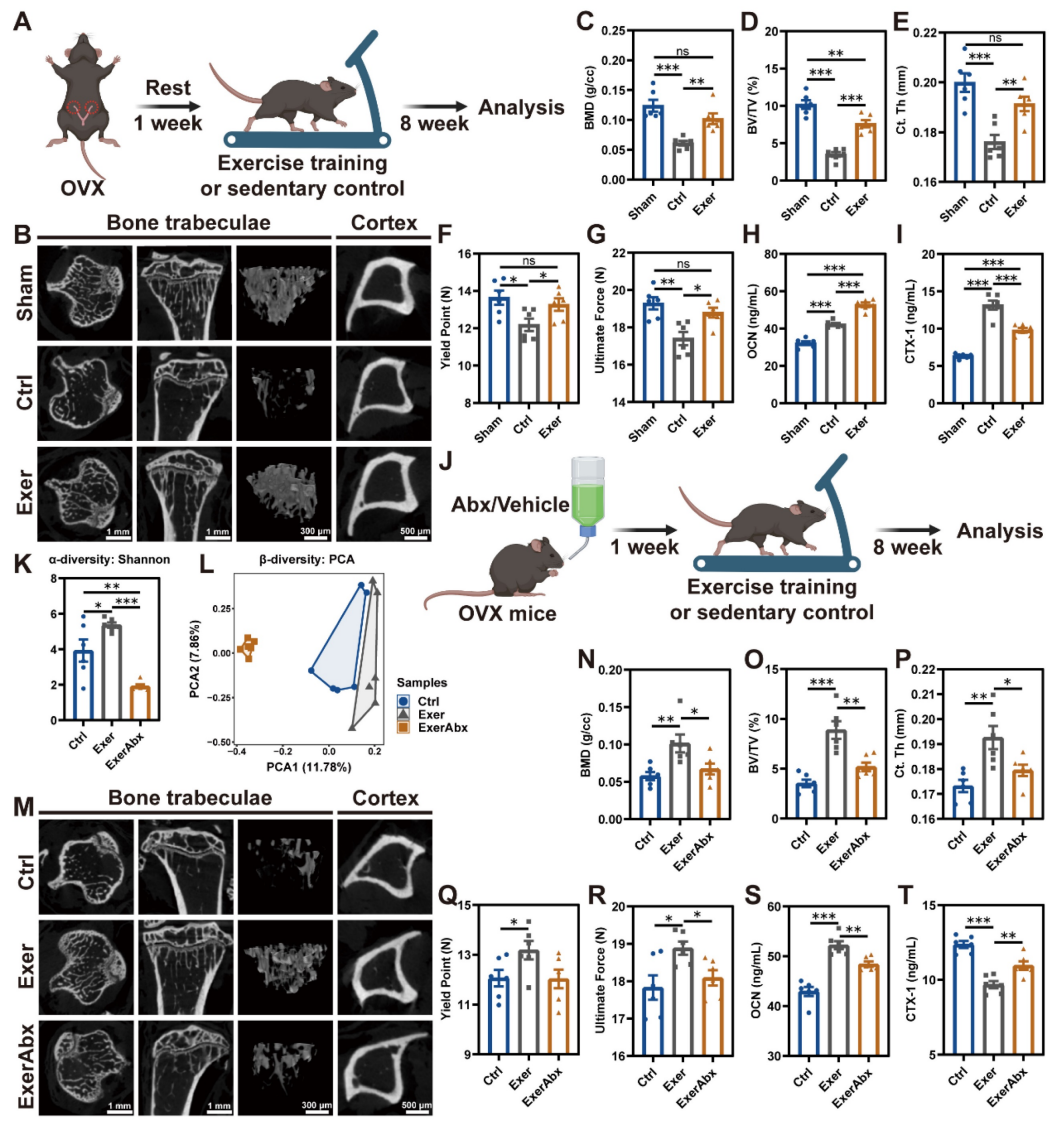
** Gut microbiota pre-depletion mitigated the protective effects of exercise against osteopenia.** (A) The schedule of exercise training after OVX. (B) Representative µCT images of trabecular and cortical bone in tibias from mice in Sham, Ctrl, and Exer groups. (C-D) Trabecular bone microarchitecture showing BMD and BV/TV. (E) Ct. Th measured in midshaft of tibias. (F-G) Biomechanical assessments of femurs showing yield point and ultimate force. (H) Serum OCN concentration. (I) Serum CTX-1 concentration. (J) The schedule of antibiotic treatment and exercise training. (K) α-diversity (Shannon index) of bacterial communities in Ctrl, Exer, and ExerAbx groups. (L) Principal component analysis (PCA) plot. (M) Representative µCT images of tibias from mice in Ctrl, Exer, and ExerAbx groups. (N) Trabecular BMD. (O) Trabecular BV/TV. (P) Ct. Th statistical results. (Q-R) Biomechanical assessments of femurs showing yield point and ultimate force. (S) Serum OCN concentration. (T) Serum CTX-1 concentration. Graphs show mean ± SEM (n = 6 per group), with statistical significance determined by one-way ANOVA followed by Bonferroni's multiple comparisons test. *P < 0.05, **P < 0.01, ***P < 0.001.

To assess the benefits of exercise training on osteopenia caused by estrogen deficiency, mice were enrolled in a treadmill exercise regimen one week post-OVX (Exer group) (Figure 1A). Sedentary mice, subjected to either sham surgery or OVX, acted as control subjects (Sham or Ctrl group). The deficiency of endogenous estrogen led to increased body weight, and physical exercise effectively reduced the OVX-induced weight gain (Figure S1A). Exercise reduced the food intake by 22% (Figure S1B), which is associated with exercise-induced anorexia 23. µCT detection demonstrated significantly decreased bone mass and microstructural deterioration in sedentary OVX mice compared to Sham mice, evidenced by substantially reduced BMD, BV/TV, Tb. N, Tb. Th, Ct. Th, and Ct. Ar/Tt. Ar, along with significantly incremental Tb. Sp (Figure 1B-E and S1C-F). Following an 8-week regimen of exercise training, these parameters of compromised bone mass and microstructures were notably restored (Figure 1B-E and S1C-F). Regular exercise training markedly enhanced the architecture of both trabecular and cortical bone. Aligned with the structural changes, the three-point bending test of femurs highlighted the improvements in biomechanical properties, with the yield point and ultimate force reflecting the bone's resistance to injury and overall integrity, respectively (Figure 1F-G). H&E staining of the proximal tibia further illustrated the augmented trabecular bone architecture attributable to physical exercise (Figure S1G). Moreover, assessment of osteoblastic and osteoclastic activities in the exercise-trained mice was conducted. IHC staining for OCN demonstrated that physical exercise promoted a substantial increment in osteoblasts along the bone surface of OVX mice (Figure S1G-H), paralleling the alterations observed in their serum OCN concentrations (Figure 1H). TRAP staining revealed that OVX precipitated a surge in osteoclast population, which was mitigated by exercise intervention (Figure S1G and S1I). The exercise regimen potently suppressed the OVX-mediated elevation of CTX-1, a serum biomarker indicative of bone resorption (Figure 1I) 24. In summary, exercise rectified bone metabolism imbalance through activation of osteoblast function and inhibition of osteoclast formation, augmenting both bone mass and strength.

To investigate whether the therapeutic effects of exercise on estrogen-deficient osteopenia depend on the gut microbiota, we pretreated mice with antibiotics (Abx) for 7 days post-OVX to deplete intestinal microbiota before initiating an 8-week exercise protocol (ExerAbx group) (Figure 1J). Mice in the other groups also received a 7-day vehicle treatment accordingly. 16S rRNA gene microbiota sequencing confirmed that exercise increased microbial richness in the gut of mice, as measured by α-diversity analysis, while Abx treatment led to a depletion of the gut microbiota (Figure 1K-L and S2A-C). Abx treatment significantly impaired the bone structure in exercised mice, manifesting as an attenuation of the osteo-protective effects of the exercise regimen (Figure 1M-P and S2D-G). The degradation of bone structure in Abx-treated mice was accompanied by increased skeletal fragility, as indicated by biomechanical assessments (Figure 1Q-R). Furthermore, Abx treatment negated the exercise-induced enhancement of osteoblast activity and suppression of osteoclastogenesis (Figure S2H-J), paralleling the alterations in serum OCN and CTX-1 levels (Figure 1S-T). These results indicated that intestinal microbiota was implicated in the exercise-induced bone-preserving action in OVX mice.

### FMT from exercised osteoporotic mice ameliorated bone loss

**Figure 2:**
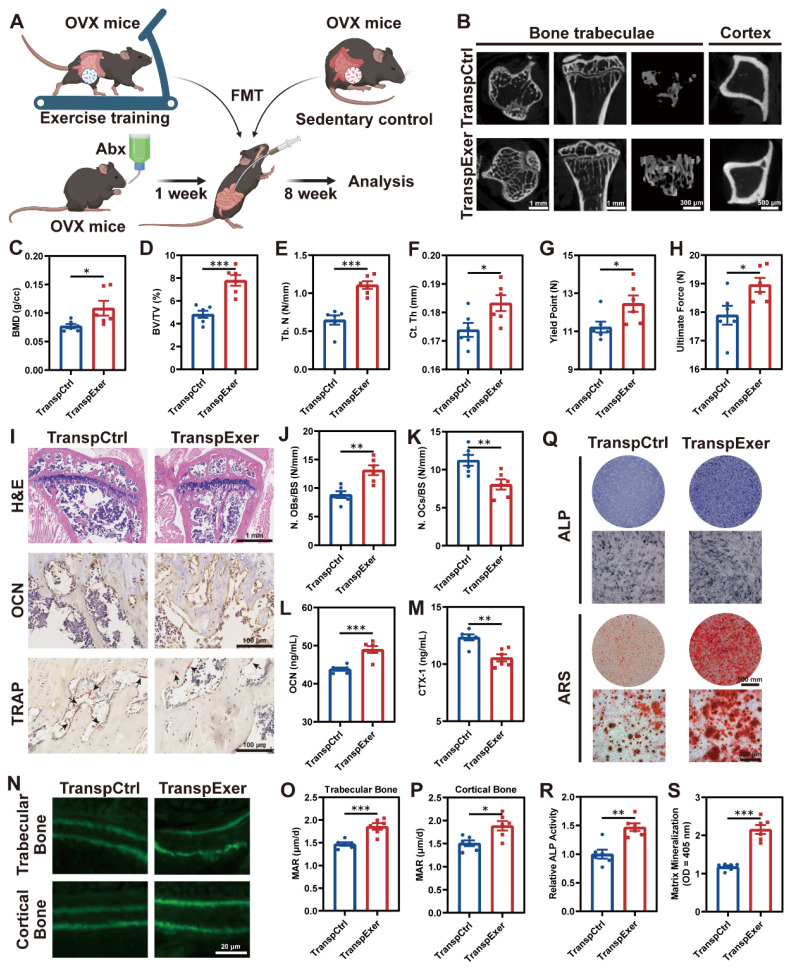
**FMT from exercised mice prevented bone loss and enhanced bone formation.** (A) The flowchart of FMT. (B) Representative µCT images of tibias from mice in TranspCtrl and TranspExer groups. (C-E) Trabecular bone microarchitecture showing BMD, BV/TV, and Tb. N. (F) Ct. Th measured in midshaft of tibias. (G-H) Biomechanical assessments of femurs showing yield point and ultimate force. (I) Representative H&E-stained, OCN-stained, and TRAP-stained sections. Black arrows indicating osteoclasts. (J) Quantitative results of number of osteoblasts per bone surface (N. OBs/BS). (K) Quantitative results of number of osteoclasts per bone surface (N. OCs/BS). (L) Serum OCN concentration. (M) Serum CTX-1 concentration. (N) Representative images of calcein double labeling. (O-P) Quantitative analyses of MAR in trabecular and cortical bones. (Q) Representative images of ALP and ARS staining of MSCs from TranspCtrl and TranspExer groups. (R) Detection of ALP activity. (S) Quantification of soluted ARS-stained nodules. Graphs show mean ± SEM (n = 6 per group), with statistical significance determined by two-tailed student *t* test. *P < 0.05, **P < 0.01, ***P < 0.001.

To elucidate the gut microbiota's role in exercise-mediated bone gain, we conducted FMT from sedentary or exercised osteoporotic mice to OVX recipients (TranspCtrl or TranspExer group) (Figure 2A). Antibiotic pretreatment was employed to create a receptive gut environment for the transplanted microbiota.

During the 8-week FMT, no noticeable variances in body weight or food intake were observed between the TranspCtrl and TranspExer groups (Figure S3A-B). Notably, mice receiving microbiota from exercised donors exhibited improved bone structure, with denser trabeculae and thicker cortical bone (Figure 2B-F and S3C-E). The enhanced bone mass and quality in the TranspExer group mice were accompanied by superior biomechanical performance, characterized by increased yield points and ultimate strength of femurs (Figure 2G-H). Furthermore, FMT from exercised OVX mice, as opposed to sedentary ones, boosted OCN^+^ osteoblasts and diminished TRAP^+^ osteoclasts on the trabecular bone surface of recipient mice (Figure 2I-K). Concurrently, serum OCN level climbed while CTX-1 level decreased in the TranspExer group (Figure 2L-M), signaling a rebalanced bone metabolism. Calcein double labeling revealed that FMT from exercised OVX mice significantly enhanced MAR in both trabecular and cortical bone, reversing the impairment of new bone formation and mineralization in osteoporotic recipients (Figure 2N-P).

Given the significant role of BM MSCs in bone homeostasis, we extracted MSCs from the BM of recipient mice and assessed cellular osteogenic potential. The TranspExer group demonstrated a marked enhancement in ALP activity, alongside increased mineralization deposits formed by BM MSCs (Figure 2Q-S). Furthermore, FMT derived from exercised OVX mice significantly upregulated the expression of osteoblast-specific differentiation genes within the recipient BM MSCs, including *Alp*, *Col1a1*, *Bglap*, *Spp1*, *Sp7*, and *Runx2* (Figure S3F-K) 25, 26. Overall, the findings indicated that the colonization of gut microbiota from exercised mice could augment the osteogenic potential of MSCs in recipients, contributing to enhanced bone formation and increased bone mass.

### FMT from exercised osteoporotic mice activated the apelin signaling pathway in MSCs

**Figure 3:**
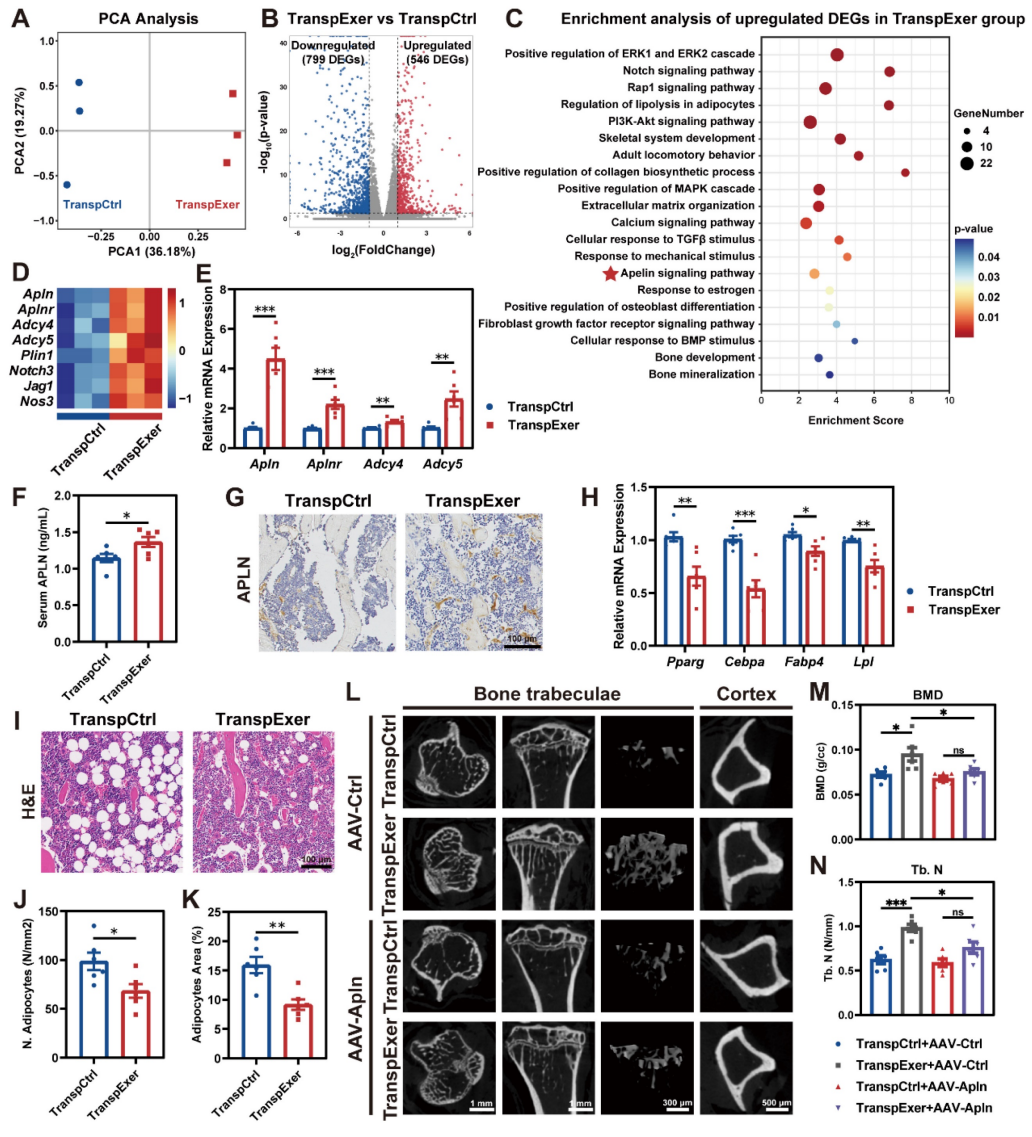
** FMT from exercised mice activated apelin signaling pathway.** (A) PCA plot of samples from TranspCtrl and TranspExer groups (n = 3 per group). (B) Volcano plot of DEGs. (C) Enrichment analysis of upregulated DEGs in the TranspExer group. (D) Heatmap of expression levels of genes associated with the apelin signaling pathway. (E) Relative mRNA expression levels of *Apln*, *Aplnr*, *Adcy4*, and *Adcy5*. (F) Serum apelin concentration. (G) Representative apelin-stained IHC sections. (H) Relative mRNA expression levels of *Pparg*, *Cebpa*, *Fabp4*, and *Lpl*. (I) Representative H&E sections depicting adipose tissue. (J-K) Quantitative analysis of J) number of adipocytes and K) adipocytes area. (L) Representative µCT images of tibias in TranspCtrl and TranspExer mice treated with AAV-Ctrl or AAV-Apln. (M-N) Trabecular bone microarchitecture showing BMD and Tb. N. Graphs show mean ± SEM (n = 6 per group), with statistical significance determined by two-tailed student *t* test in E-F, H, and J-K, and one-way ANOVA followed by Bonferroni's multiple comparisons test in M-N. *P < 0.05, **P < 0.01, ***P < 0.001.

To elucidate the molecular mechanisms underlying the bone gain of FMT from exercised osteoporotic mice, we harvested BM MSCs from OVX mice following FMT and executed transcriptomic profiling. PCA indicated that MSCs from the TranspExer group and the TranspCtrl group displayed distinct transcriptomic landscapes (Figure 3A). Compared to the TranspCtrl group, MSCs from the TranspExer mice showed a marked shift in gene expression, with 546 genes significantly upregulated and 799 genes downregulated (Figure 3B). Enrichment analysis of upregulated DEGs following the exercise-matched FMT identified the activation of key pathways, including mitogen-activated protein kinase/extracellular signal-regulated kinase (MAPK/ERK) 27, Notch 28, RAS-proximate-1 (Rap1) 29, phosphoinositide-3-kinase and protein kinase B (PI3K-AKT) 30, transforming growth factor beta (TGF-β) 31, and calcium signaling pathways 32, all of which are important for osteogenic differentiation and align with the previously demonstrated osteogenic traits (Figure 3C).

Beyond the canonical osteogenic pathways, certain lipid metabolism pathways were upregulated following the exercise-matched FMT, including regulation of lipolysis and apelin signaling pathway (Figure 3C-D). Notably, apelin, a key exerkine, has been reported to repress adipogenesis and facilitate bone mass augmentation 33, 34. Subsequent RT-qPCR validation further confirmed the upregulation of *Apln*, *Aplnr*, *Adcy4*, and *Adcy5* in MSCs from the TranspExer group (Figure 3E). Serum assays and bone IHC staining verified the elevated systemic and skeletal apelin levels in recipient mice treated with the exercise-matched FMT (Figure 3F-G). Given that extensive research has validated the adipogenesis activation and the bone-fat balance disruption as significant drivers of skeletal aging 35, 36, the adipogenic genes in FMT-treated mice were further assessed. We observed a significant reduction in the mRNA expression levels of adipogenic markers (*Pparg*, *Cebpa*, *Fabp4*, and *Lpl*) in the MSCs from the TranspExer group (Figure 3H). Furthermore, histological assessment of tibial sections revealed a significant decrease in both adipocyte quantity and size within the TranspExer group, indicative of attenuated adipogenesis (Figure 3I-K). Overall, FMT from exercised osteoporotic mice activated the apelin signaling pathway, promoted osteogenesis, and inhibited adipogenesis in MSCs of recipient OVX mice.

In order to elucidate the function of the apelin signaling pathway in the bone gain resulting from the exercise-matched FMT, the apelin receptor blocker ML221 was used to interfere with apelin signaling. Following the treatment with ML221, the augmentation of bone mass triggered by the exercise-matched FMT was suppressed (Figure S4A-H). Besides, ML221 downregulated osteogenic genes (*Alp* and *Runx2*) and upregulated adipogenic genes (*Pparg* and *Lpl*) in MSCs from the TranspExer mice (Figure S4I-J). Moreover, we validated the important role of apelin by knocking it down with AAV encapsulating apelin shRNA (AAV-Apln). After injection of AAV-Apln, EGFP fluorescence was detected *via* live imaging (Figure S4K), and both serum APLN level and *Apln* gene expression in MSCs significantly decreased (Figure S4L-M). Similar to the effects of ML221 treatment, AAV-Apln significantly inhibited the bone gain of the exercise-matched FMT on OVX mice (Figure 3L-N and S4N-T). These results implied a pivotal role for the apelin signaling pathway in the bone benefits associated with exercise-induced FMT.

Enrichment analysis of DEGs revealed the activation of inflammatory pathways in the TranspCtrl group, including cellular responses to IL1 and IFNγ, as well as IL17 and TNF signaling pathways (Figure S5A-B). Besides, the TranspCtrl group exhibited substantial upregulation in aging and replicative senescence (Figure S5A-B). Estrogen insufficiency is implicated in the dysregulation of inflammatory responses, thereby accelerating aging and bone loss 37. To elucidate the impact of FMT in this scenario, we assessed inflammatory factor levels within the BM. As shown in Figure S5C-G, the exercise-matched FMT significantly decreased the levels of inflammatory factors within the BM of osteoporotic mice. These findings indicated that the microbiota from exercised mice could profoundly mitigate inflammation in the BM of OVX mice, fostering a bone homeostasis-friendly microenvironment.

### Exercise and optimized gut microbiota reinforced intestinal barrier and inhibited inflammation

**Figure 4:**
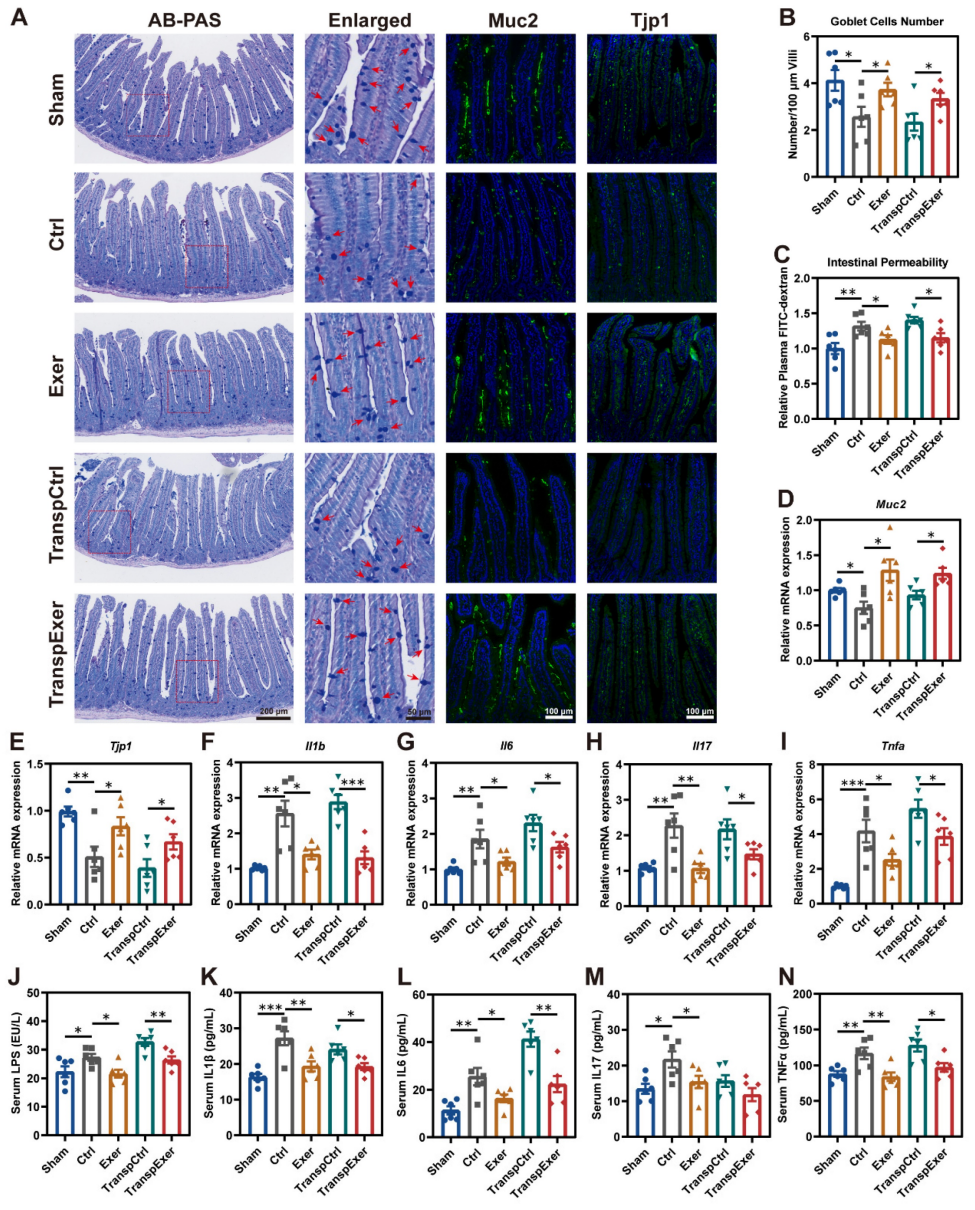
** Exercise and corresponding FMT protected intestinal barrier and reduced inflammation in OVX mice.** (A) Representative images showing intestinal structure by AB-PAS staining and immunofluorescence staining of Muc2 and Tjp1. Red arrows indicating goblet cells. (B) Quantitative results of goblet cells. (C) The intestinal permeability measured by circulating FITC-labeled dextran. (D-E) The mRNA expression levels of genes associate with intestinal barrier, including *Muc2* and *Tjp1*. (F-I) The mRNA expression levels of inflammatory factors (*Il1b*, *Il6*, *Il17*, and *Tnfa*) in small intestinal tissue. (J) Serum LPS concentration. (K-N) Serum levels of inflammatory factors, including IL1β, IL6, IL17, and TNFα. Graphs show mean ± SEM (n = 6 per group), with statistical significance determined by one-way ANOVA followed by Bonferroni's multiple comparisons test. *P < 0.05, **P < 0.01, ***P < 0.001.

As the intestine is the primary site for microbiota colonization and their impact on the host, we examined its biological functions and characteristics to elucidate the effects of physical training and microbial transplantation on the intestine. Goblet cells are specialized epithelial cells that secrete mucus to provide a protective barrier for the intestine 38, and we observed that both exercise and exercise-matched FMT substantially enhanced the population of goblet cells (Figure 4A-B). Moreover, the rise in circulating FITC-labeled dextran levels pointed to enhanced intestinal permeability due to estrogen depletion, which could be mitigated by both exercise and its corresponding gut microbiota (Figure 4C). Further immunofluorescent staining of Muc2 and Tjp1 in intestinal tissue supported the beneficial effect of exercise and related FMT in barrier integrity (Figure 4A), which was consistent with the upregulation of related gene expression (Figure 4D-E). We also observed similar manifestations in the colon (Figure S6), suggesting that exercise and exercise-matched FMT effectively improved the intestinal mucosal barrier 39, 40.

Previous studies have confirmed that intestinal barrier dysfunction precipitates microbiota and metabolic disturbances, culminating in inflammation and expedited bone loss 41, 42. Thus, intestinal barrier integrity may affect bone metabolism through inflammatory signaling. RT-qPCR demonstrated that OVX surgery dramatically elevated gene expression levels of critical inflammatory factors (*Il1b*, *Il6*, *Il17*, and *Tnfa*) in the small intestine, which were ameliorated by exercise and corresponding FMT (Figure 4F-I). Disruption of the intestinal barrier may lead to the entry of bacterial toxic products into the bloodstream 43, so we measured circulating LPS levels, which were found to be consistent with changes of intestinal permeability (Figure 4J). Moreover, exercise and corresponding FMT significantly reduced inflammatory factor levels in serum (Figure 4K-N), potentially accounting for the decreased inflammation within the BM (Figure S5C-G).

### Exercise training modified the gut microbiota with transmissible traits

**Figure 5:**
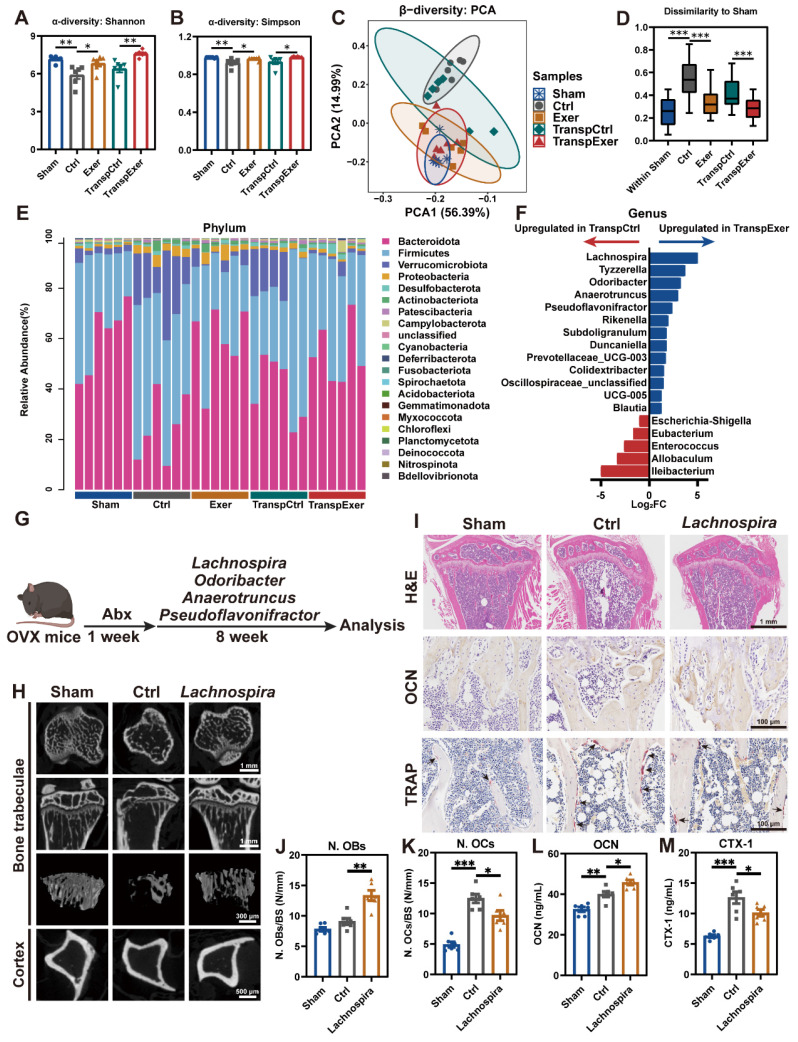
** Exercise training modified the gut microbiota with transmissible traits.** (A-B) The results of α-diversity (Shannon and Simpson index) of bacterial communities in Sham, Ctrl, Exer, TranspCtrl, and TranspExer groups. (C) PCA plot based on microbial composition. (D) Quantitative analysis of UniFrac dissimilarity among the five groups. (E) Fecal bacterial community at the phylum level among samples. (F) Differential microbiota between TranspCtrl and TranspExer mice at the genus level. (G) Flowchart of the gavage procedure for key probiotics in OVX mice. (H) Representative µCT images of tibias from mice in Sham, Ctrl, and *Lachnospira* groups. (I) Representative H&E-stained, OCN-stained, and TRAP-stained sections. Black arrows indicating osteoclasts. (J-K) Quantitative results of N. OBs/BS and N. OCs/BS. (L-M) Serum levels of OCN and CTX-1. Graphs show mean ± SEM (n = 6 per group), with statistical significance determined by Wilcoxon rank test in F, and one-way ANOVA followed by Bonferroni's multiple comparisons test in other graphs. *P < 0.05, **P < 0.01, ***P < 0.001.

To elucidate the relevance between the exercise-induced bone protection and the gut microbiome, we assessed the bacterial taxonomic profiles in fecal samples by 16S rRNA gene sequencing. α-diversity analysis (Shannon and Simpson index) revealed that OVX surgery induced a decrease in gut microbiota richness, which was reversed by exercise, aligning with the changes shown in the respective FMT recipient groups (Figure 5A-B). β-diversity assessment showed distinct gut microbiota profiles across groups, with sham-operated and exercised OVX mice exhibiting greater similarity to each other (Figure 5C). Quantitative UniFrac dissimilarity analysis further confirmed that the gut microbiota of the Exer and TranspExer groups resembled that of the Sham group, signifying the successful execution of the FMT strategy (Figure 5D).

LEfSe analysis was utilized to identify distinct bacterial taxa among the various groups (Figure S7A). Fecal sample analysis at the phylum level revealed substantial alterations in the microbial composition (Figure 5E and S7B). At the genus level, hierarchical clustering analysis of groups revealed a striking similarity between donor mice and corresponding recipients, with the Exer and TranspExer groups exhibiting a marked affinity with the Sham group (Figure S7C-D). Differentially abundant features analysis at the genus level revealed a significant augmentation of *Ileibacterium*, *Allobaculum*, *Enterococcus*, *Eubacterium*, and *Escherichia-Shigella* in the TranspCtrl group (Figure 5F). Conversely, the TranspExer group demonstrated a significant enrichment of various probiotic genera, including *Lachnospira*, *Tyzzerella*, *Odoribacter*, *Anaerotruncus*, *Pseudoflavonifractor*, *Rikenella*, *Subdoligranulum*, and *Duncaniella* (Figure 5F). Collectively, these findings implied that the microbiota from exercised osteoporotic mice successfully transmitted and reshaped the intestinal microbiota profiles of the recipient OVX mice.

To identify the critical microbial communities responsible for bone protective effects during FMT, several probiotics (*Lachnospira*, *Odoribacter*, *Anaerotruncus*, and *Pseudoflavonifractor*), abundant in the intestinal tracts of TranspExer mice, were administered *via* gavage as a therapeutic intervention for OVX mice (Figure 5G). The radiological findings indicated that administration of *Lachnospira* significantly enhanced the trabecular bone quality and cortical bone thickness in OVX mice, while other probiotics yielded weaker effects on bone mass (Figure 5H and S7E-L). Mechanical tests further confirmed that *Lachnospira* enhanced the load-bearing capacity of bone (Figure S7M-N). In the tibial tissue of *Lachnospira*-treated OVX mice, the bone microarchitecture was effectively maintained (Figure 5I), which was associated with increased osteoblast activity and suppressed osteoclast formation (Figure 5J-K). Correspondingly, following the treatment with* Lachnospira*, the serum level of OCN significantly increased, while the level of CTX-1 significantly decreased (Figure 5L-M). Overall, *Lachnospira* was likely responsible for the bone-protective effects during the transfer of gut ecological traits, and supplementing with *Lachnospira* could delay the progression of osteoporosis in estrogen-deficient mice.

### The bile acid metabolism pathway mediated the bone gain from exercise-matched FMT

**Figure 6:**
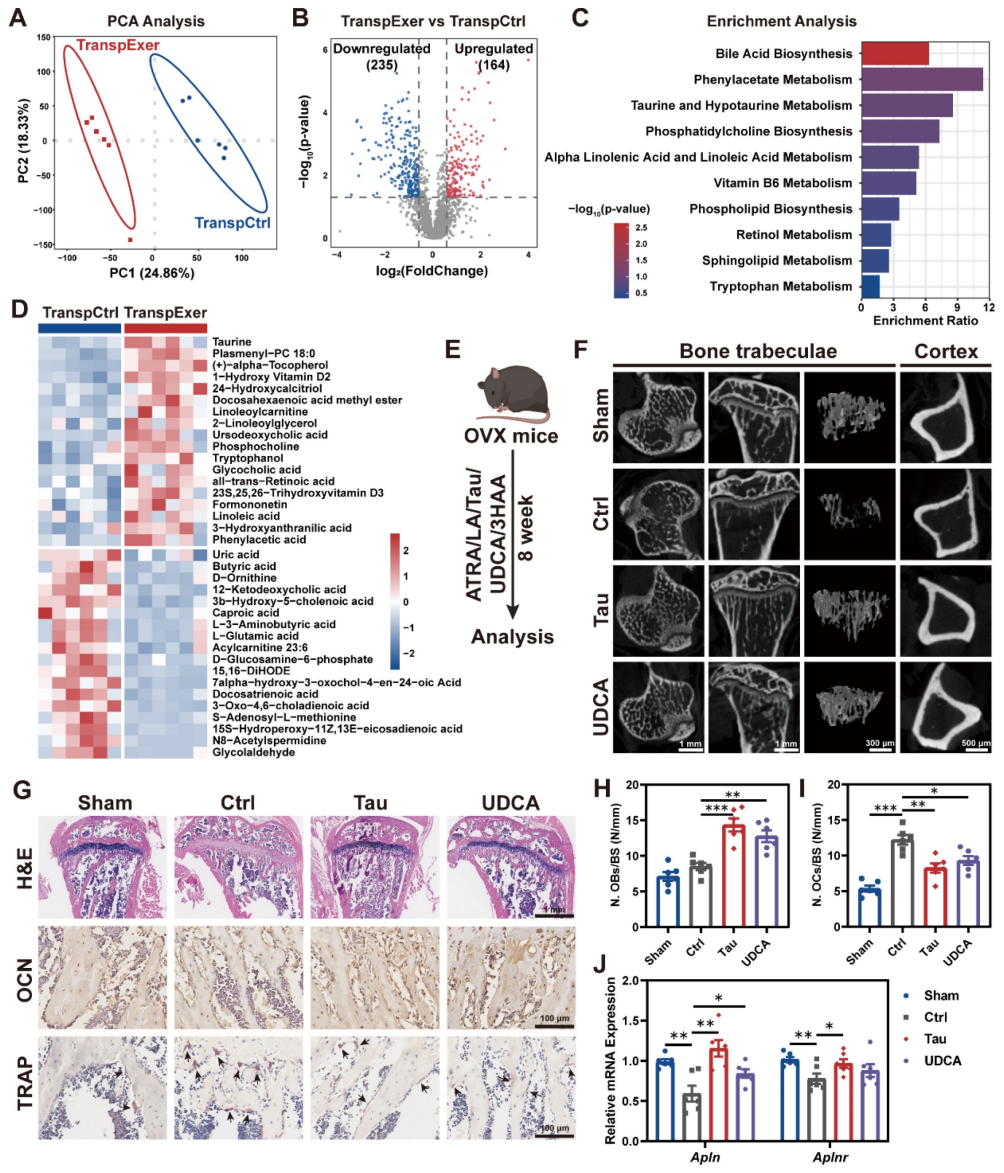
** Exercise-matched FMT reshaped the gut metabolite profiles in OVX mice.** (A) PCA plot. (B) Volcano plot of differential metabolites. (C) Enrichment analysis of upregulated metabolites in the TranspExer group. (D) Heatmap of differential metabolites between the TranspCtrl and TranspExer groups. (E) Flowchart of the gavage procedure for key metabolites in OVX mice. (F) Representative µCT images of tibias from mice in Sham, Ctrl, Tau, and UDCA groups. (G) Representative H&E-stained, OCN-stained, and TRAP-stained sections. Black arrows indicating osteoclasts. (H-I) Quantitative results of N. OBs/BS and N. OCs/BS. (J) Relative RNA expression levels of genes *Apln* and *Aplnr*. Graphs show mean ± SEM (n = 6 per group), with statistical significance determined by one-way ANOVA followed by Bonferroni's multiple comparisons test. *P < 0.05, **P < 0.01, ***P < 0.001.

Emerging evidence has implicated gut microbiota-derived metabolites as influential in the pathogenesis of osteoporosis 44, 45. To ascertain the specific metabolites that confer the exercise-induced protection of bone mass, we applied LC-MS untargeted metabolomic sequencing to fecal specimens and observed significant differences of gut metabolite profiles between the TranspExer and TranspCtrl groups (Figure 6A-B). Enrichment analysis of the metabolites upregulated in the TranspExer group revealed the significant activation of pathways, including bile acid biosynthesis pathway (encompassing taurine and hypotaurine metabolism), alpha linolenic acid and linoleic acid metabolism, retinol metabolism, and tryptophan metabolism (Figure 6C). To identify metabolites accounting for the bone benefits of exercise-matched FMT, OVX-induced osteoporosis mouse models were applied to screen metabolites based on our metabolome sequencing data (Figure 6D). Several metabolites that were upregulated in TranspExer group were identified as candidate metabolites, including Tau, UDCA, LA, ATRA, and 3HAA.

Following the OVX surgery in mice, we initiated a supplement protocol of daily gavage with these metabolites for eight weeks (Figure 6E). The μCT analysis of tibias revealed that Tau and UDCA significantly mitigated the estrogen deficiency-induced damage to trabecular microarchitecture and cortical bone loss (Figure 6F), while ATRA, LA, and 3HAA had no discernible impact on bone mass alterations (Figure S8A-H). Besides, long-term supplementation with Tau and UDCA markedly improved the biomechanical strength of femurs, as evidenced by increased yield point and ultimate force (Figure S8I-J). Parallel to imaging results, histological examination of the proximal tibias in Tau and UDCA-treated mice revealed the enhancement of bone microarchitecture in the marrow, coupled with elevated osteoblastic activity and diminished osteoclastogenesis (Figure 6G-I). Consistently, serum biomarkers confirmed a marked rise in the osteogenic marker OCN and a significant decline in the osteoclastic marker CTX-1 (Figure S8K-L), indicative of a restoration of bone homeostasis.

To investigate whether key metabolites recapitulate the effects of exercise-matched FMT, we evaluated the transcriptional profiling of BM MSCs. As shown in Figure 6J, both Tau and UDCA significantly upregulated apelin signaling pathway-related genes (*Alpn* and *Alpnr*). Besides, there was a marked upregulation of osteogenic markers (*Alp*, *Runx2*, *Spp1*, and *Sp7*) and a concurrent downregulation of adipogenic genes (*Pparg* and *Lpl*) in MSCs from mice treated with Tau or UDCA (Figure S8M-O), indicating a pro-osteogenic and anti-adipogenic shift. Collectively, these findings suggested that the exercise-matched FMT significantly modified the gut microbiota and metabolite profiles in OVX mice, serving as a potential mechanism for preserving bone mass. The alternations of bile acid metabolism profiles, indicated by the enhanced enrichment of Tau and UDCA, is believed to play an important role in the exercise-induced protective effects on bone health.

### Exercise-matched FMT and pivotal metabolites ameliorated age-related bone loss

**Figure 7:**
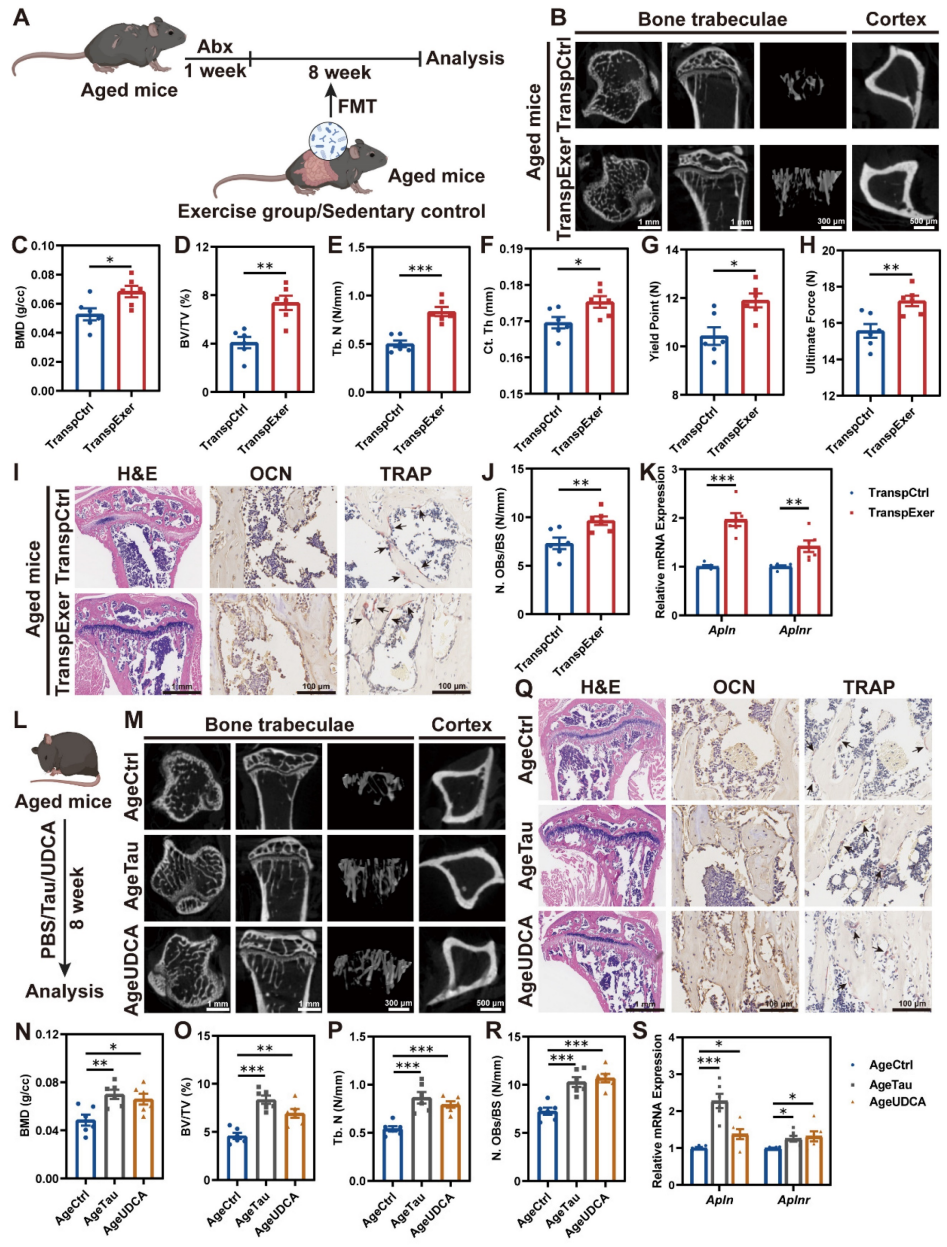
** Exercise-matched FMT and key metabolites ameliorated age-related bone loss.** (A) Flowchart of FMT administration in aged mice. (B) Representative µCT images of tibias from aged mice in the TranspCtrl group and the TranspExer group. (C-E) Trabecular bone microarchitecture showing C) BMD, D) BV/TV, and E) Tb. N. (F) Ct. Th statistical results. (G-H) Biomechanical results of G) yield point and H) ultimate force. (I) Representative H&E-stained, OCN-stained, and TRAP-stained sections from the TranspCtrl group and the TranspExer group. Black arrows indicating osteoclasts. (J) Quantitative results of N. OBs/BS. (K) Relative mRNA expression levels of genes *Apln* and *Aplnr*. (L) Flowchart of metabolites supplementation in aged mice. (M) Representative µCT images of tibias from aged mice in the AgeCtrl, AgeTau, and AgeUDCA groups. (N-P) Trabecular bone microarchitecture showing N) BMD, O) BV/TV, and P) Tb. N. (Q) Representative H&E-stained, OCN-stained, and TRAP-stained sections from the AgeCtrl, AgeTau, and AgeUDCA groups. Black arrows indicating osteoclasts. (R) Quantitative results of N. OBs/BS. (S) Relative mRNA expression levels of genes *Apln* and *Aplnr*. Graphs show mean ± SEM (n = 6 per group), with statistical significance determined by two-tailed student *t* test in C-H and J-K, and one-way ANOVA followed by Bonferroni's multiple comparisons test in N-S. *P < 0.05, **P < 0.01, ***P < 0.001.

Estrogen deficiency serves as a pivotal driver of bone loss in postmenopausal women, yet human epidemiological studies and animal mechanism research indicate that aging with increased systemic inflammation may be another vital contributor to bone metabolism disorder 46, 47. Therefore, we further assessed the impact of exercise-matched FMT on bone mass in aged mouse models (Figure 7A). The trabecular microstructure in aged mice was found to be sparse, whereas microbial transplantation from exercised aged mice significantly enhanced trabecular thickness and density, as well as cortical bone thickness (Figure 7B-F and S9A-C). Fragility fractures, common among the elderly, are linked to bone's biomechanical traits 48. Aged mice in the TranspExer group exhibited significantly greater yield points and ultimate forces compared to the TranspCtrl group, indicating a marked enhancement in mechanical robustness (Figure 7G-H). H&E and OCN staining of the tibias illustrated that the increased bone mass in the TranspExer group was accompanied by heightened osteoblast activity, with TRAP staining indicating no significant alterations in osteoclastogenesis (Figure 7I-J and S9D).

To examine the impact of FMT on MSCs in the BM of aged mice, we evaluated the osteogenic potential and mineral deposition capabilities of MSCs. The TranspExer group demonstrated enhanced ALP activity and increased biomineralization (Figure S9E-G), along with a robust induction of osteogenic genes expression (*Alp*, *Runx2*, *Spp1*, and *Sp7*) (Figure S9H). Simultaneously, FMT from exercised aged mice downregulated adipogenesis-related genes (*Pparg* and *Lpl*) (Figure S9I), which was previously reported to be elevated in aged MSCs 49. Additionally, the genes *Apln* and* Aplnr* were significantly upregulated in the TranspExer group (Figure 7K). Taken together, the exercise-matched FMT might trigger the apelin pathway and reoriented differentiation path in MSCs from aged mice, thereby reversing the age-induced osteoporosis.

Investigating the potential of the bile acid metabolism pathway, highlighted by Tau and UDCA, in reversing skeletal aging following FMT from exercised elderly mice is intriguing. To this end, we administered Tau and UDCA to aged mice for 8 weeks and subsequently analyzed the skeletal effects (Figure 7L). In μCT analysis, Tau and UDCA significantly enhanced bone microarchitecture and cortical thickness of tibias from aged mice, especially in the AgeTau group (Figure 7M-P and S9J-M). Concurrently, there was a discernible upturn in the mechanical robustness of femurs from the Tau and UDCA-treated mice (Figure S9N-O), signifying an encompassing rejuvenation of skeletal integrity and performance. In alignment with the benefits of exercise-matched FMT, Tau and UDCA supplementation effectively stimulated bone formation through increasing osteoblast activity (Figure 7Q-R and S9P). RT-qPCR profiling of BM-derived MSCs from Tau and UDCA-treated aged mice revealed the activation of the apelin signaling pathway (Figure 7S), with a significant upregulation of osteogenic differentiation and a concurrent suppression of adipogenesis-related gene expression (Figure S9Q-R). Collectively, Tau and UDCA emerged as potential inhibitors of skeletal aging and contributed to exercise-induced bone health preservation.

## Discussion

In this study, we explored the role of the gut microbiota in exercise-mediated protection against osteoporosis through employing sophisticated multi-omics approaches, including transcriptomic sequencing, 16S rRNA gene sequencing, and LC-MS untargeted metabolomics analysis. Our findings demonstrated that both physical activity and associated FMT improved gut health and barrier integrity. Moreover, FMT from exercised osteoporotic mice effectively activated apelin signaling pathway, alleviated systemic inflammation, and delayed the onset of osteoporosis in estrogen-deficient recipients. The gut bile acid profile in TranspExer mice exhibited a significant shift, characterized by an increase in Tau and UDCA, strongly involved in enhanced bone mass. The therapeutic potentials of exercise-matched FMT and key metabolites were further validated in aged mice. Together, the gut microbiota and corresponding metabolites from exercised mice contributed to the bone gain of exercise.

The interaction effects between gut microbiota and bone metabolism have been the focus of extensive study for many years 50-52. Individuals with osteoporosis, induced by estrogen deficiency or aging, exhibited significant alterations in their gut microbiota profiles 51. Compared to wild-type mice, germ-free mice showed higher bone mass and lower osteoclast activity 53, 54. Interestingly, transplantation of gut microbiota from children, rather than the elderly, could attenuate the OVX-induced osteoporotic phenotype 17. Our research reinforced the growing consensus that the gut microbiota mediates the bone benefits of exercise. Physical training has been broadly recognized as a key non-pharmacological strategy against osteoporosis, known to regulate gut microbiota and increase the abundance of probiotics 55, 56, in line with the findings of our study. However, the regulatory role and underlying mechanisms of exercise in the gut-bone axis remain unknown. Our findings offered strong evidence that gut-bone crosstalk plays a pivotal role in the exercise-mediated therapy of osteopenia, and the gut microbiota and metabolites from exercised mice showed promise in counteracting bone loss resulted from estrogen deficiency or aging. Treadmill exercise is a common form of aerobic exercise for modern individuals, and it serves as the basis for the exercise model in this study. It is worth noting that different exercise models, including aerobic exercise, resistance training, and high-intensity interval training (HIIT), may exert distinct physiological effects 57-59. Investigating these variations is crucial for further exploration in subsequent studies to fully understand the implications of various exercise types on the intricate relationship within the microbiome-metabolite-bone axis.

The intestinal tract harbors a diverse microbial community, and disturbances in this biodiversity are associated with the onset of various diseases 60, 61. Notably, estrogen insufficiency can precipitate intestinal lining injury, heighten permeability, and incite an inflammatory cascade, thereby contributing to the progression of osteoporosis 62, 63. Our study demonstrated that eight weeks following OVX, the murine intestinal tract manifested severe pathological alterations characterized by compromised barrier integrity, heightened inflammation, and reduced microbial richness, while these adverse effects could be effectively counteracted by a regimen of physical exercise and corresponding FMT. Moreover, the gastrointestinal supplementation of *Lachnospira* was found to delay the progress of osteoporosis in OVX mice. *Lachnospira* supplementation has been shown to facilitate gastrointestinal repair and mitigate inflammation following radiation exposure 64. Overall, the microbiota derived from exercised mice significantly improved the gut ecosystem of the recipients.

The gut microbiota is conceptualized as a novel endocrine organ, capable of directly generating or governing the synthesis of diverse bioactive metabolites, which influence the physiological functions of distant organ systems 61, 65. To delineate the potential mechanisms underlying the bone gain mediated by the exercise-altered gut microbiota, we utilized LC-MS untargeted metabolomics to identify a multitude of key metabolites. Our data revealed a substantial enrichment of Tau and UDCA in the intestines of the TranspExer group, indicative of activated bile acid metabolism and alterations in the bile acid profile. Subsequent oral administration of Tau and UDCA to mice demonstrated potent inhibitory effects on the exacerbation of bone loss attributable to aging or OVX. As key signaling molecules, bile acids play a critical role in inter-organ communication, with their profile characteristics being closely linked to health and disease states 66, 67. It has been reported that Tau deficiency is considered as a driver of aging, and supplementation of Tau could slow organ senescence 68, 69. UDCA, predominantly used for chronic liver conditions 70, possesses anti-inflammatory properties and is not yet fully explored 71. We underscored the critical role of the intestinal bile acid profile in the gut-bone axis. As pivotal metabolites, Tau and UDCA hold substantial promise for enhancing skeletal well-being and preventing fragile fractures, thereby offering expansive potential for clinical interventions.

The disruption of the bone-fat balance resulted from aging or estrogen deficiency, characterized by inhibited bone formation and activated adipogenesis, plays an important role in osteopenia 72, 73. It is widely believed that the benefits of mechanical loading on skeletal health are achieved by restoring the bone-fat balance 36. We employed transcriptome sequencing to analyze the gene expression of BM MSCs, with the objective of elucidating the molecular mechanisms by which the exercise-matched FMT attenuates the progression of osteoporosis. The functional enrichment analysis of DEGs upregulated in the TranspExer group revealed the upregulation of the apelin signaling pathway, a crucial exercise-responsive mechanism 33. Several studies have suggested that apelin promotes osteogenesis and suppresses adipogenesis, yet this signaling pathway remains underexplored 34, 74. Our experimental results confirmed that the intestinal microbiota from exercised mice activated the apelin signaling pathway, enhanced osteoblast activity, and suppressed adipogenesis. Furthermore, in osteoporotic mice supplemented with Tau and UDCA, we also observed a rise in apelin-associated gene expression. Collectively, the activation of the apelin signaling pathway and the restoration of the bone-fat balance were integral to the bone gain from the exercise-matched FMT.

In summary, our study revealed that exercise mitigated osteoporosis progression in mice through the gut microbiota-mediated bile acid metabolism pathway based on multi-omics analysis. By modulating the intestinal environment or supplementing therapeutic agents, the bone-protective effects of exercise could be replicated to serve those unable to engage in physical activity. As insights into the interplay between intestinal microecology and bone metabolism deepen, the gut-bone axis is emerging as a novel therapeutic avenue for osteoporosis 50, 51. Our research broadens this perspective, highlighting the potential of gut microbiota and their metabolites as a promising strategy for osteoporosis prevention and treatment.

## Supplementary Material

Supplementary figures and table.
