# Supplementary material for: Exercise ameliorates osteopenia in mice via intestinal microbial-mediated bile acid metabolism pathway
Source: Theranostics. 2025 Jan 2;15(5):1741–59. doi: 10.7150/thno.104186 (PMC11780523; doi:10.7150/thno.104186)
Supplement: Supplementary file 1 — Supplementary figures and table. [file thnov15p1741s1.pdf]

Supplementary Material

Exercise ameliorates osteopenia in mice via intestinal microbial-mediated bile acid metabolism pathway

Congcong Yu[a,b,#], Rongtai Sun[a,b,#], Wentao Yang[a,b], Tianyuan Gu[a,b], Xiaozhang Ying[c], Lin Ye[a,b], Yang Zheng[a,b,d], Shunwu Fan[a,b,*], Xiangjun Zeng[e,*], and Shasha Yao[a,b,*]

[a]Department of Orthopaedic Surgery, Sir Run Run Shaw Hospital School of Medicine, Zhejiang University, Hangzhou, Zhejiang 310016, China

[b]Key Laboratory of Musculoskeletal System Degeneration and Regeneration, Translational Research of Zhejiang Province Hangzhou, Zhejiang 310016, China

[c]Zhejiang Hospital of Integrated Traditional Chinese and Western Medicine, Hangzhou, Zhejiang 310016, China

[d]Research Institute of Orthopedics, The Affiliated Jiangnan Hospital of Zhejiang Chinese Medical University, Hangzhou, Zhejiang 310053, China

[e]Bone Marrow Transplantation Center of the First Affiliated Hospital & Liangzhu Laboratory, Zhejiang University School of Medicine, Hangzhou, Zhejiang 311100, China

[#]These authors have contributed equally.

[*]Correspondence: yaoshasha@zju.edu.cn (S. Yao), xjzeng@zju.edu.cn (X. Zeng), shunwu_fan@zju.edu.cn (S. Fan)

Table S1. Primers used in RT-qPCR

Gene	Forward primer (5' to 3')	Reverse primer (5' to 3')
Gapdh	AGGTCGGTGTGAACGGATTTG	TGTAGACCATGTAGTTGAGGTCA
Alp	TGACCTTCTCTCCTCCATCC	CTTCCTGGGAGTCTCATCCT
Col1a1	GCTCCTCTTAGGGGCCACT	CCACGTCTCACCATTGGGG
Bglap	CTTGAAGACCGCCTACAAAC	GCTGCTGTGACATCCATAC
Spp1	AGCAAGAAACTCTTCCAAGCAA	GTGAGATTCGTCAGATTCATCCG
Sp7	ATGGCGTCCTCTCTGCTTG	TGAAAGGTCAGCGTATGGCTT
Runx2	TTCAACGATCTGAGATTTGTGGG	GGATGAGGAATGCGCCCTA
Apln	CACTGATGTTGCCTCCAGATGGA	ACGCCATTAGACGAACTTGGTGG
Aplnr	GGTTACAACTACTATGGGGCTGA	AGCTGAGCGTCTCTTTTCGC
Adcy4	AGTACCCACTGCTGATACTGC	AGCCACCCAAAGCACACAG
Adcy5	CTTGGGGAGAAGCCGATTCC	ACCGCTTAGTGGAGGGTCT
Pparg	TCGCTGATGCACTGCCTATG	GAGAGGTCCACAGAGCTGATT
Cebpa	CAAGAACAGCAACGAGTACCG	GTCACTGGTCAACTCCAGCAC
Fabp4	AAGGTGAAGAGCATCATAACCCT	TCACGCCTTTCATAACACATTCC
Lpl	GGGAGTTTGGCTCCAGAGTTT	TGTGTCTTCAGGGGTCCTTAG
Muc2	ATGCCCACCTCCTCAAAGAC	GTAGTTTCCGTTGGAACAGTGAA
Cdh1	CAGGTCTCCTCATGGCTTTGC	CTTCCGAAAAGAAGGCTGTCC
Tjp1	GCCGCTAAGAGCACAGCAA	TCCCCACTCTGAAAATGAGGA
Il1b	GCAACTGTTCCTGAACTCAACT	ATCTTTTGGGGTCCGTCAACT
Il6	TAGTCCTTCCTACCCCAATTTCC	TTGGTCCTTAGCCACTCCTTC
Il17	TTTAACTCCCTTGGCGCAAAA	CTTTCCCTCCGCATTGACAC
Tnfa	CCCTCACACTCAGATCATCTTCT	GCTACGACGTGGGCTACAG

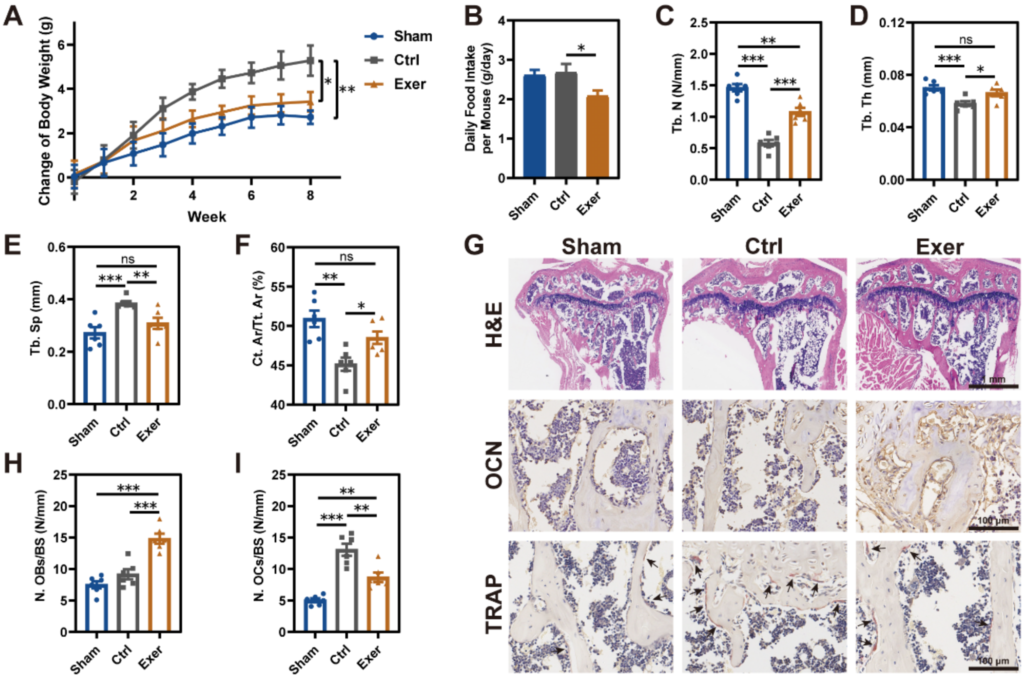

Figure S1. Exercise training improved bone mass in OVX mice. Related to Figure 1. (A) Body weight changes of mice in the Sham, Ctrl, and Exer groups over 8 weeks. (B) Statistical results of daily food intake. (C-E) Trabecular bone microarchitecture showing Tb. N, Tb. Th, and Tb. Sp. (F) Ct. Ar/Tt. Ar measured in midshaft of tibias. (G) Representative H&E-stained, OCN-stained, and TRAP-stained sections. (H) Quantitative results of N. OBs/BS. Black arrows indicating osteoclasts. (I) Quantitative results of N. OCs/BS. Graphs show mean ± SEM (n = 6 per group), with statistical significance determined by one-way ANOVA followed by Bonferroni's multiple comparisons test. *P < 0.05, **P < 0.01, ***P < 0.001.

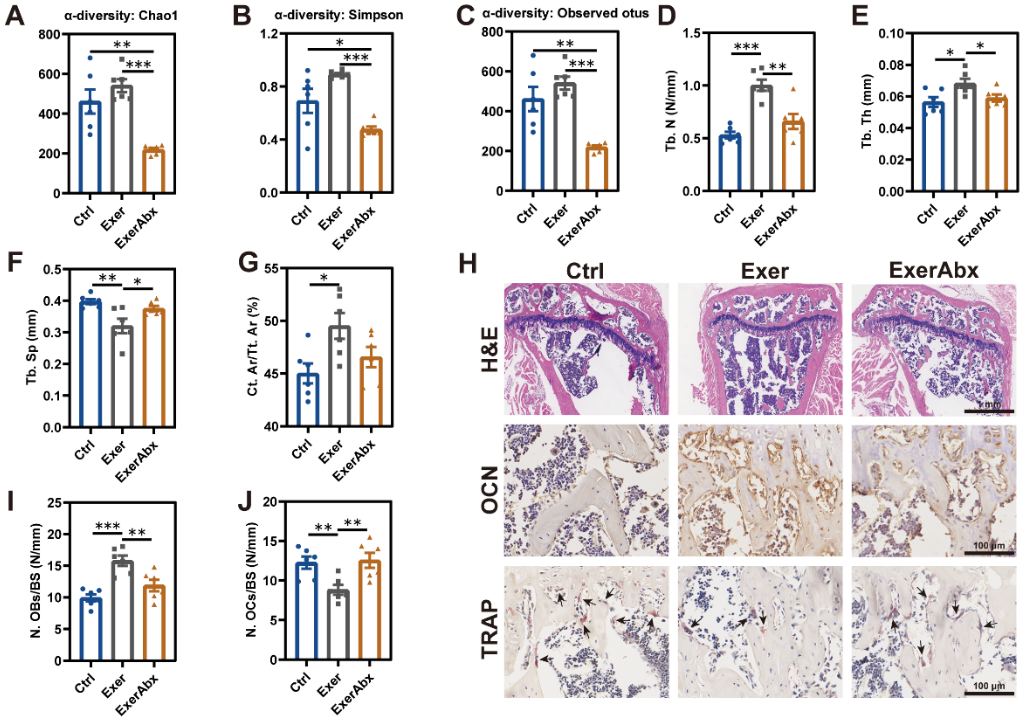

Figure S2. Gut microbiota pre-depletion mitigated the protective effects of exercise against osteopenia. Related to Figure 1. (A-C) α-diversity (Chao1, Simpson, and Observed otus index) of bacterial communities in Ctrl, Exer, and ExerAbx groups. (D-F) Trabecular bone microarchitecture showing Tb. N, Tb. Th, and Tb. Sp. (G) Ct. Ar/Tt. Ar measured in midshaft of tibias. (H) Representative H&E-stained, OCN-stained, and TRAP-stained sections. Black arrows indicating osteoclasts. (I) Quantitative results of N. OBs/BS. (J) Quantitative results of N. OCs/BS. Graphs show mean ± SEM (n = 6 per group), with statistical significance determined by one-way ANOVA followed by Bonferroni's multiple comparisons test. *P < 0.05, **P < 0.01, ***P < 0.001.

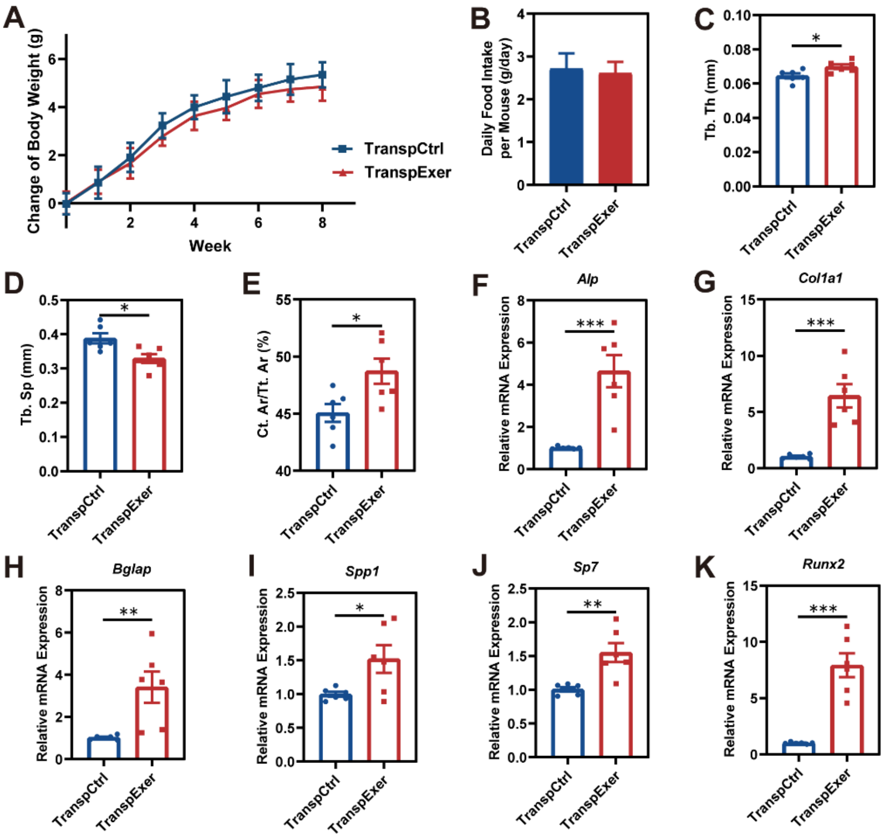

Figure S3. FMT from exercised mice prevented bone loss and enhanced bone formation. Related to Figure 2. (A) Body weight changes of mice in the TranspCtrl, and TranspExer groups over 8 weeks. (B) Statistical results of daily food intake. (C-D) Trabecular bone microarchitecture showing Tb. Th and Tb. Sp. (E) Ct. Ar/Tt. Ar measured in midshaft of tibias. (F-K) Relative gene expression levels of osteogenic markers, including *Alp*, *Col1a1*, *Bglap*, *Spp1*, *Sp7*, and *Runx2*. Graphs show mean ± SEM (n = 6 per group), with statistical significance determined by two-tailed student *t* test. *P < 0.05, **P < 0.01, ***P < 0.001.

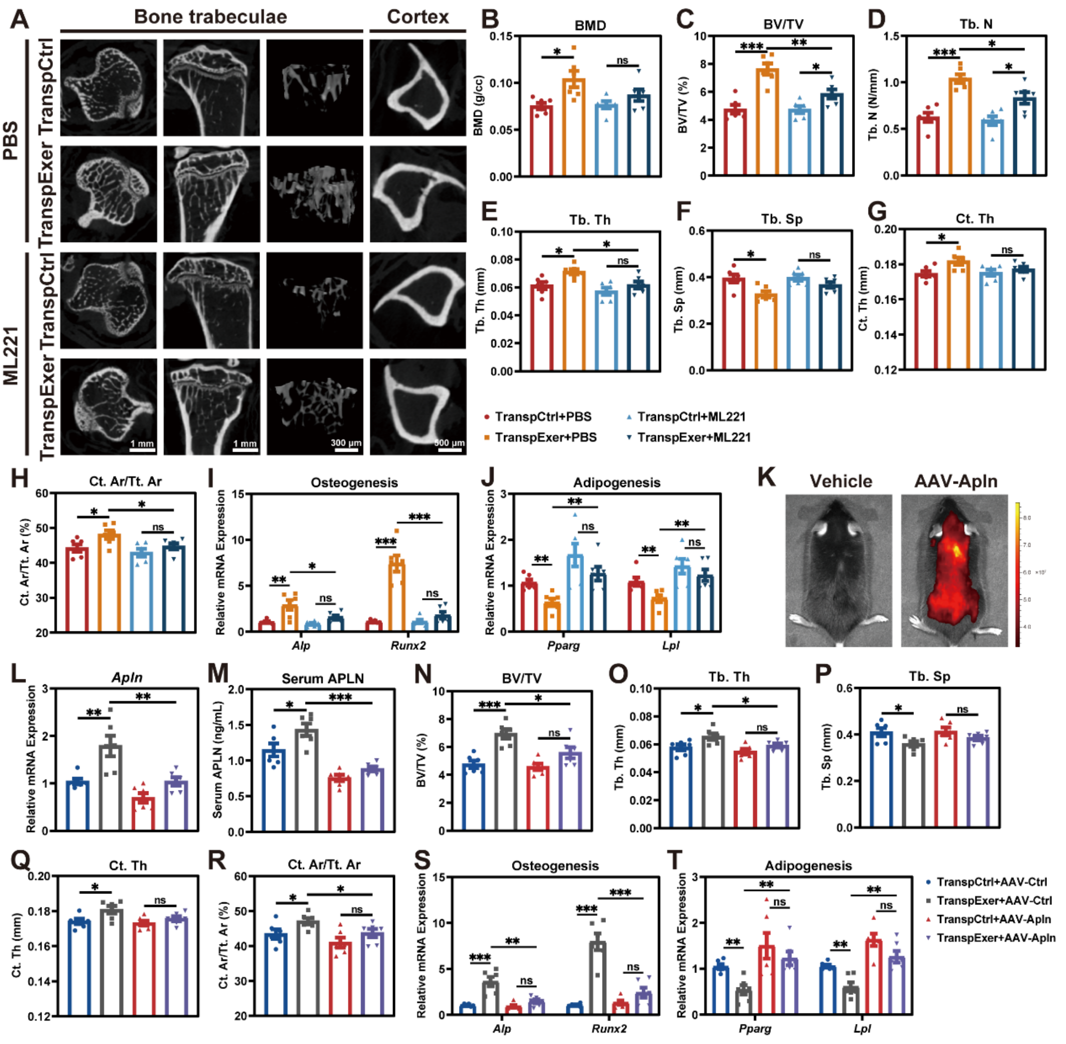

Figure S4. FMT from exercised mice activated apelin signaling pathway. Related to Figure 3. (A) Representative μCT images of tibias in TranspCtrl and TranspExer mice treated with PBS or ML221. (B-F) Trabecular bone microarchitecture showing BMD, BV/TV, Tb. N, Tb. Th, and Tb. Sp. (G-H) Ct. Th and Ct. Ar/Tt. Ar measured in midshaft of tibias. (I) Relative mRNA expression levels of *Alp* and *Runx2*. (J) Relative mRNA expression levels of *Pparg* and *Lpl*. (K) Fluorescence expression of the co-transfected EGFP in mice after tail vein injection of AAV-Apln. (L) Relative mRNA expression of *Apln* in MSCs. (M) Serum APLN level. (N-P) Trabecular bone microarchitecture showing BV/TV, Tb. Th, and Tb. Sp. (Q-R) Ct. Th and Ct. Ar/Tt. Ar measured in midshaft of tibias. (S) Relative mRNA expression levels of *Alp* and *Runx2*. (T) Relative mRNA expression levels of *Pparg* and *Lpl*. Graphs show mean ± SEM (n = 6 per group), with statistical significance determined by one-way ANOVA followed by Bonferroni's multiple comparisons test. *P < 0.05, **P < 0.01, ***P < 0.001.

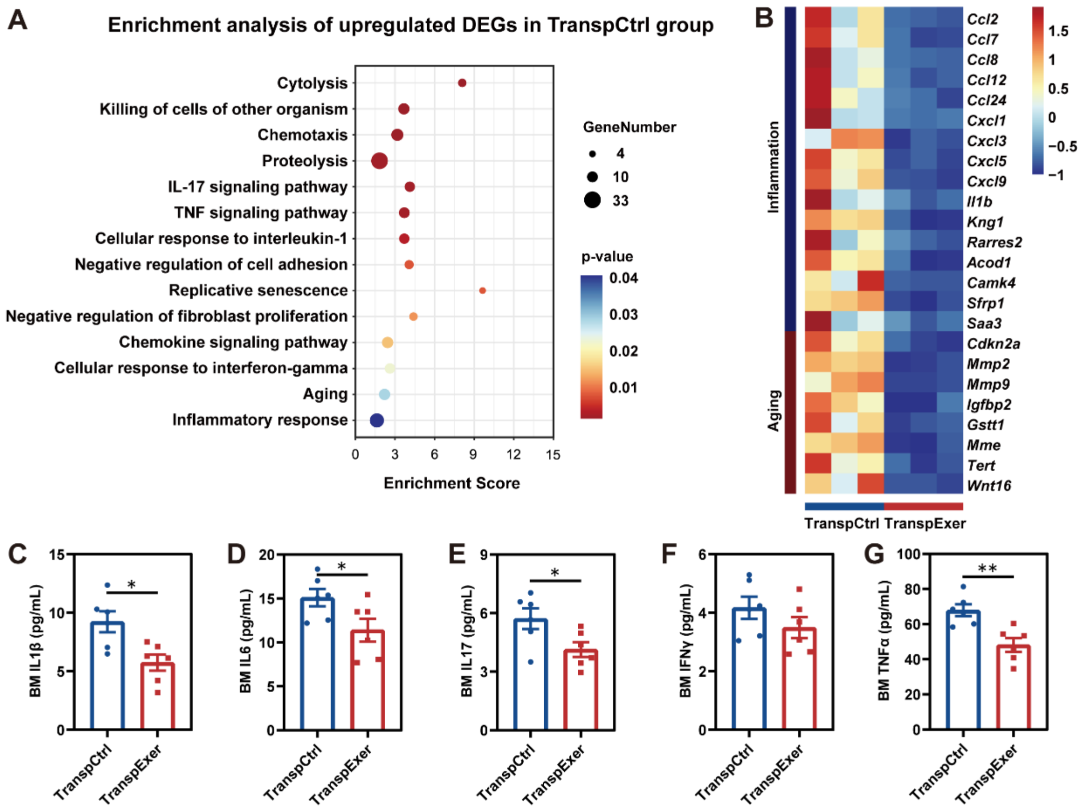

Figure S5. FMT from exercised mice suppressed inflammation. Related to Figure 3. (A) Enrichment analysis of upregulated DEGs in the TranspCtrl group. (B) Heatmap of expression levels of genes associated with inflammation and aging. (C-G) IL1β, IL6, IL17, IFNγ, and TNFα levels in the BM. Graphs show mean ± SEM (n = 6 per group), with statistical significance determined by two-tailed student *t* test. *P < 0.05, **P < 0.01.

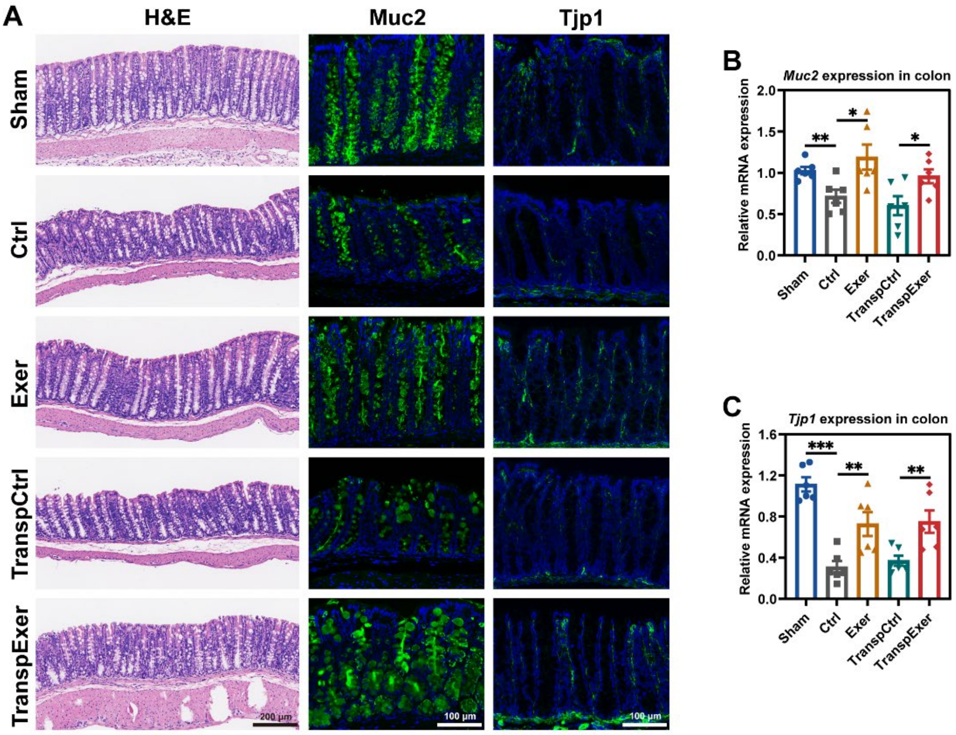

Figure S6. Exercise and corresponding FMT protected intestinal barrier and reduced inflammation in OVX mice. Related to Figure 4. (A) Representative images showing colon structure by H&E staining and immunofluorescence staining of Muc2 and Tjp1. (B-C) The mRNA expression levels of genes associate with intestinal barrier, including *Muc2* and *Tjp1*. Graphs show mean ± SEM (n = 6 per group), with statistical significance determined by one-way ANOVA followed by Bonferroni's multiple comparisons test. *P < 0.05, **P < 0.01, ***P < 0.001.

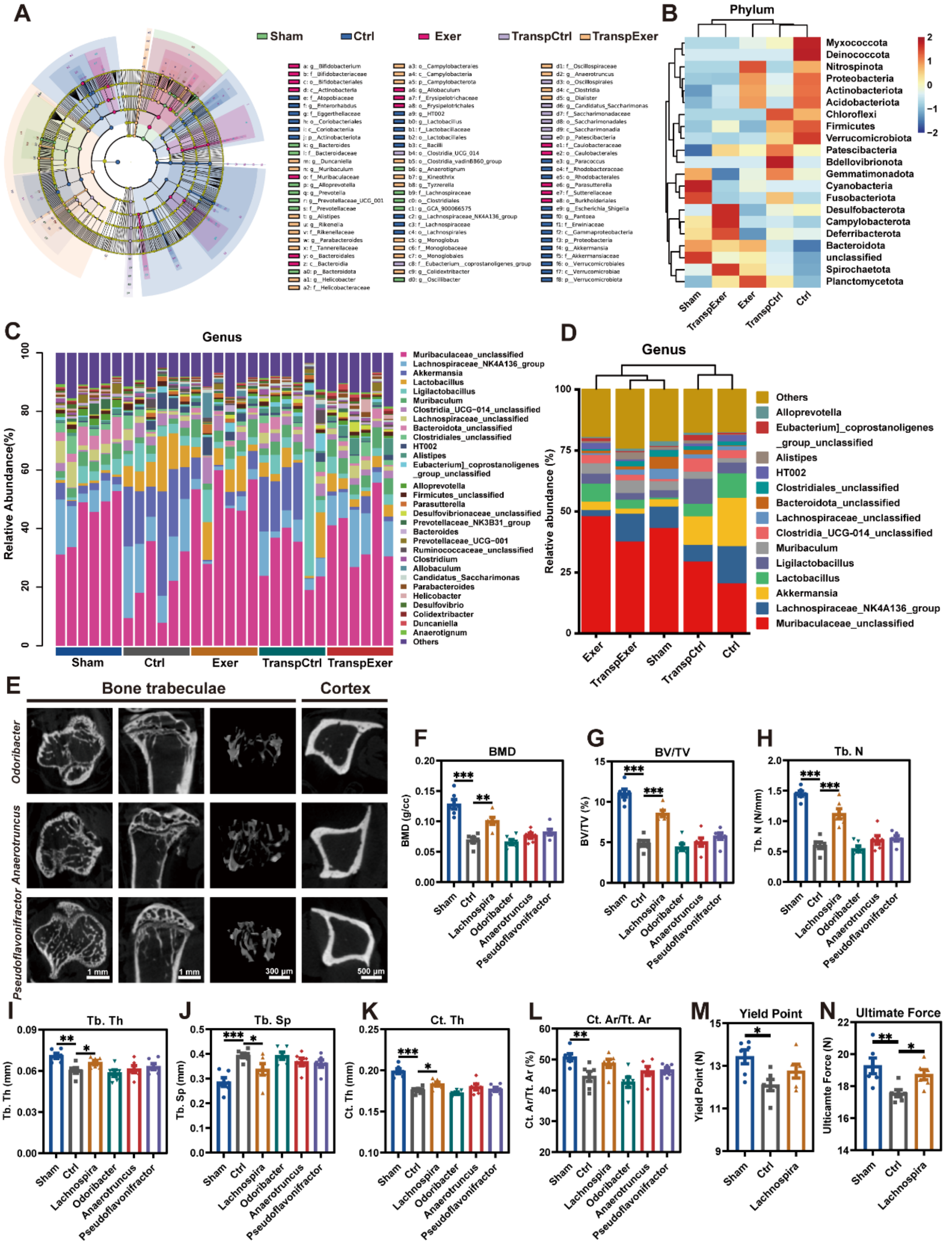

Figure S7. Exercise training modified the gut microbiota with transmissible traits. Related to Figure 5. (A) Taxonomic cladogram obtained from LEfSe analysis showing differential bacterial taxa among Sham, Ctrl, Exer, TranspCtrl, and TranspExer groups. (B) Average relative abundance of prevalent microbiota at the phylum level among five groups. (C) Relative abundance of microbiota at the genus level among five groups. (D) Average relative abundance of bacterial community at the genus level among the five

groups. (E) Representative μCT images of tibias from mice in *Odoribacter*, *Anaerotruncus*, and *Pseudoflavonifractor* groups. (F-J) Trabecular bone microarchitecture showing BMD, BV/TV, Tb. N, Tb. Th, and Tb. Sp. (K-L) Ct. Th and Ct. Ar/Tt. Ar measured in midshaft of tibias. (M-N) Biomechanical results of yield point and ultimate force. Graphs show mean ± SEM (n = 6 per group), with statistical significance determined by one-way ANOVA followed by Bonferroni's multiple comparisons test. *P < 0.05, **P < 0.01, ***P < 0.001.

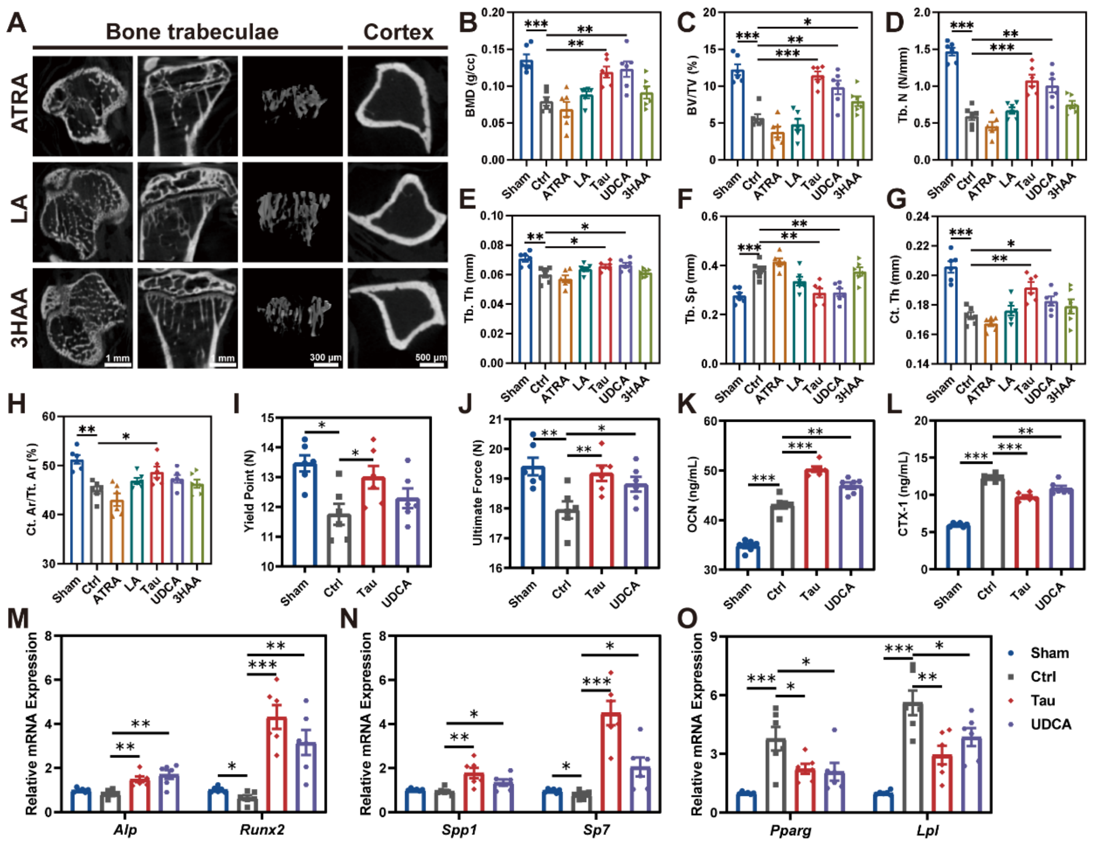

Figure S8. Exercise-matched FMT reshaped the gut metabolite profiles in OVX mice. Related to Figure 6. (A) Representative μCT images of trabecular and cortical bone in ATRA, LA, and 3HAA groups. (B-F) Trabecular bone microarchitecture showing BMD, BV/TV, Tb. N, Tb. Th, and Tb. Sp. (G-H) Statistical results of Ct. Th and Ct. Ar/Tt. Ar. (I-J) Biomechanical results of yield point and ultimate force. (K-L) Serum OCN and CTX-1 concentration. (M-N) Relative mRNA expression levels of osteogenic markers (*Alp*, *Runx2*, *Spp1*, and *Sp7*). (O) Relative mRNA expression levels of adipogenic markers (*Pparg* and *Lpl*). Graphs show mean ± SEM (n = 6 per group), with statistical significance determined by one-way ANOVA followed by Bonferroni's multiple comparisons test. *P < 0.05, **P < 0.01, ***P < 0.001.

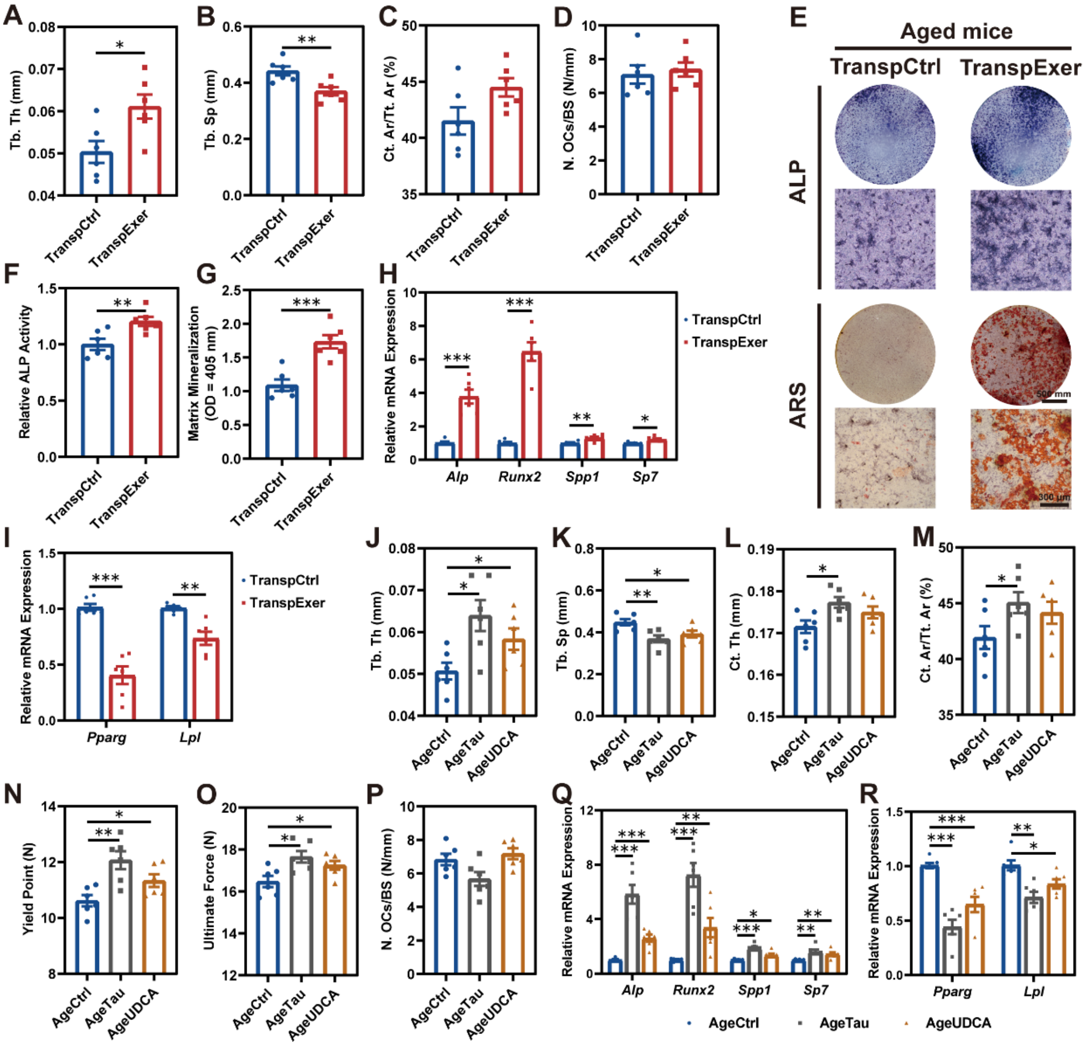

Figure S9. Exercise-matched FMT and key metabolites ameliorated age-related bone loss. Related to Figure 7. (A-B) Trabecular bone microarchitecture showing Tb. Th and Tb. Sp. (C) Statistical results of Ct. Ar/Tt. Ar. (D) Quantitative results of N. OCs/BS. (E) Representative images of ALP and ARS staining of MSCs from aged mice in the TranspCtrl and TranspExer groups. (F) Detection of ALP activity. (G) Quantification of soluted ARS-stained nodules. (H) Relative mRNA expression levels of osteogenic markers (*Alp*, *Runx2*, *Spp1*, and *Sp7*). (I) Relative mRNA expression levels of adipogenic markers (*Pparg* and *Lpl*). (J-K) Trabecular bone microarchitecture showing Tb. Th and Tb. Sp. (L-M) Statistical results of Ct. Th and Ct. Ar/Tt. Ar. (N-O) Biomechanical results of yield point and ultimate force. (P) Quantitative results of N. OCs/BS. (Q) Relative mRNA expression levels of osteogenic markers (*Alp*, *Runx2*, *Spp1*, and *Sp7*). (R) Relative mRNA expression levels of adipogenic markers (*Pparg*

and *Lpl*). Graphs show mean ± SEM (n = 6 per group), with statistical significance determined by two-tailed student *t* test in A-D and F-I, and one-way ANOVA followed by Bonferroni's multiple comparisons test in J-R. *P < 0.05, **P < 0.01, ***P < 0.001.
